# Green Space Quality and Health: A Systematic Review

**DOI:** 10.3390/ijerph182111028

**Published:** 2021-10-20

**Authors:** Phi-Yen Nguyen, Thomas Astell-Burt, Hania Rahimi-Ardabili, Xiaoqi Feng

**Affiliations:** 1 School of Population Health, University of New South Wales, Sydney, NSW 2033, Australia; phiyen.nguyen@unsw.edu.au (P.-Y.N.); h.rahimiardabili@unsw.edu.au (H.R.-A.); 2 Population Wellbeing and Environment Research Lab (PowerLab), Wollongong, NSW 2522, Australia; thomasab@uow.edu.au; 3 School of Health and Society, Faculty of Arts, Social Sciences, and Humanities, University of Wollongong, Wollongong, NSW 2522, Australia

**Keywords:** green space qualities, parks, streetscape greenery, environmental types, built environment, physical health, mental health, cardiovascular diseases, respiratory diseases, quality of life

## Abstract

(1) Background: As cities densify, researcher and policy focus is intensifying on which green space types and qualities are important for health. We conducted a systematic review to examine whether particular green space types and qualities have been shown to provide health benefits and if so, which specific types and qualities, and which health outcomes. (2) Methods: We searched five databases from inception up to June 30, 2021. We included all studies examining a wide range of green space characteristics on various health outcomes. (3) Results: 68 articles from 59 studies were found, with a high degree of heterogeneity in study designs, definitions of quality and outcomes. Most studies were cross-sectional, ecological or cohort studies. Environment types, vegetation types, and the size and connectivity of green spaces were associated with improved health outcomes, though with contingencies by age and gender. Health benefits were more consistently observed in areas with greater tree canopy, but not grassland. The main outcomes with evidence of health benefits included allergic respiratory conditions, cardiovascular conditions and psychological wellbeing. Both objectively and subjectively measured qualities demonstrated associations with health outcomes. (4) Conclusion: Experimental studies and longitudinal cohort studies will strengthen current evidence. Evidence was lacking for needs-specific or culturally-appropriate amenities and soundscape characteristics. Qualities that need more in-depth investigation include indices that account for forms, patterns, and networks of objectively and subjectively measured green space qualities.

## 1. Introduction

Green spaces are a crucial aspect of urban cities. They protect against many of the harmful impacts of rapid urbanisation on health. They also permit social and economic benefits by providing preferential settings for relaxation, building social connections, engaging in physical activity and feeling closer to nature, including resident wildlife [1]. Therefore, urban greening is an important strategy for addressing complex global issues such as climate change, sustainable urbanisation and health inequality. This is recognised via the United Nations Sustainable Development Goal (SDG) 11 target 7, which states “by 2030, providing universal access to safe, inclusive and accessible, green and public spaces, in particular for women and children, older persons and persons with disabilities” [2].

Substantial research is dedicated to revealing the health benefits of green spaces [3]. While more green space tends to be good for health, such conclusions are not universally reported. Most research in this field tends to use measures of ‘greenness’ such as the normalised difference vegetation index (NDVI) to quantify green space exposure [4], ignoring substantial heterogeneity in the constituent qualities of green spaces that make them attractive for visiting and, in turn, support health and wellbeing. For example, green spaces may vary in terms of objectively measurable good qualities (e.g., presence of certain attractive elements, such as tree canopy, footpaths and seating) and others that are more subjective in nature (e.g., an emotional or spiritual connection to a particular green space). Bad qualities (e.g., proximity to a busy road and lack of accessibility) may discourage visitation and negate health benefits. Ignoring the constituent qualities that attract or discourage people to spend time in green spaces holds back the field from having more substantive impacts as a catalyst for improving community health and reducing inequities. Examining these qualities, both good and bad, may solve a missing link in our understanding of the relationship between green spaces and health [5].

Moreover, studying green space qualities has practical implications for urban planning. Driven by rapid densification, the compact, high-density city has become the dominant urban design worldwide. Not only does a compact city warrant multifunctional green spaces that can serve its diverse citizen population. It also presents a complex set of trade-offs between green space creation, regeneration and expansion on one hand, and the development of new, often competing land-use on the other (e.g., housing, infrastructure and commercial) [6]. Within space constrained contexts, modifying qualities of existing green spaces may offers an important way to maintain and improve quality of life in urban communities.

Research on the health benefits of green space qualities is still emerging and there are no consensus definition what green space quality is. We do not know which qualities can be modified, and which health benefits these modifications will bring (if any). To build capacities for research that attends to these issues, we conducted a systematic review to take stock of what research has been performed on green space qualities and health, with the broader aim of charting possible paths forward to strengthen the policy relevance of this research.

This systematic review aims to:

(a) Evaluate whether improving certain qualities of green space provides health benefits to the population;
(b) Identify and categorise all qualities of green space that have been investigated in previous primary studies; and
(c) Explore the extent of variations in design characteristics of these studies.

## 2. Materials and Methods

The reporting of this review was guided by the updated Preferred Reporting Items for Systematic Reviews and Meta-Analyses (PRISMA) guideline [7]. This review was not registered a priori, nor was a protocol published separately.

### 2.1. Search Strategy

We searched the following databases for articles from inception up to 8 December 2020: MEDLINE via Ovid, Embase via Ovid, PsycINFO via Ovid, CINALH via EBSCO and Scopus. No language or publication date restriction was applied. An updated search was performed on 30 June 2021. The search was supplemented by a manual search of the reference lists from relevant systematic reviews.

The search strategy was a combination of three components: (health outcomes AND green space quality AND green space types). For health outcomes, we used both generic and specific search terms to capture all dimensions of physical and mental health, drawing from previous systematic literature reviews on green space and health [8,9], obesity and physical activity [10,11], birth outcomes [12], mental health [13,14,15], puberty timing [16] and menopause [17]. For green space quality, we combined the word “quality” and other determinant terms adapted from audit tools used for assessing the physical environment of parks [18]. For green space types, we used both generic and specific search terms to capture all types of green space in both urban and rural settings. The full search strategy is available in Supplementary File S1.

### 2.2. Study Selection

We included all human studies meeting the following criteria:

(a) Population: green space users of all ages and genders;
(b) Exposure: In the context of our review, green space quality refers to any attribute that can affect willingness to use and interaction of users with that space, including but not limited to intrinsic characteristics (size or patterns), features (vegetation, facilities or amenities), conditions (maintenance or safety) or user perception of its usefulness or quality. All types of natural and man-made green environments, including parks, streetscape greenery, urban open spaces, playgrounds, coastal parks with vegetation, etc., were included as long as they were defined by authors as green space. Studies where participants viewed digitalised renderings or photographs of green spaces without actual exposure were excluded. Studies that did not investigate any aspect of green space quality were excluded. The percentage of overall vegetation coverage and “greenness” (e.g., the normalised difference vegetation index) were not eligible as they are considered measures of green space quantity, unless specific vegetation types were analysed (e.g., tree canopy);
(c) Outcomes: Studies that investigated health outcomes, including but not limited to cardiometabolic, respiratory, reproductive, neurological and psychological health, and child development, were included. Studies that only measured behaviours (park usage, park-based activity, etc.) without assessing health outcomes were excluded;
(d) Study design: All observational and intervention studies, including randomised, quasi-randomised and non-randomised trials. We excluded non-English language studies, study protocols, conference abstracts, dissertations, reviews, qualitative studies, editorials, case studies and opinion pieces.

All retrieved data were imported into Covidence (Veritas Health Innovation, Australia) to remove duplicates. Two reviewers (PYN and HR-A) independently screened all titles and abstracts in duplicate and excluded studies that did not meet the inclusion criteria. Studies that were included from title/abstract screening had their full text reviewed in duplicate by the same two reviewers and reasons for exclusion were noted. Disagreement was resolved by discussion with senior reviewers (XF and TA-B). All stages of study screening were conducted in Covidence. 

### 2.3. Data Extraction and Quality Appraisal

One reviewer (P-YN) extracted the data using a standard data extraction form and a second reviewer (HR-A) validated 10% of the studies for accuracy. The data extracted included: study characteristics (location, time, settings), population’s demographic and clinical characteristics, green space types, green space quality domains, health outcomes and corresponding measures of association. We also recorded the tools used to assess green space quality and health outcomes, effect measures reported, types of statistical analyses conducted and any adjustment for confounding factors. Based on the effect measures and 95% confidence intervals, we recorded the direction of effect for each study, i.e., whether the study presented some evidence of protective associations, some evidence of risk associations, or no significant associations at all.

One reviewer (P-YN) appraised the methodological quality of all included studies using the quality assessment tools for the appropriate study types [19] and the second reviewer (HR-A) validated 10% for accuracy. Because these tools do not provide for ecological studies, the existing tool for observational cohort and cross-sectional studies were adapted by adding 3 criteria addressing ecological fallacy, spatial autocorrelation and uncertainty in fitting spatial data [20,21]. Based on the list of applicable criteria, each study was given an adjusted quality score of 0–10 (Supplementary File S2). Disagreement was resolved with consensus via discussion with senior reviewers (XF and TA-B), if required. 

### 2.4. Data Analysis

We used inductive categorisation to develop a set of domains of green space quality based on definitions reported in the included studies and stratified the findings of the studies based on these quality domains. Due to the heterogeneity of exposure, intervention and outcomes, meta-analysis was not conducted.

## 3. Results

**Figure 1:**
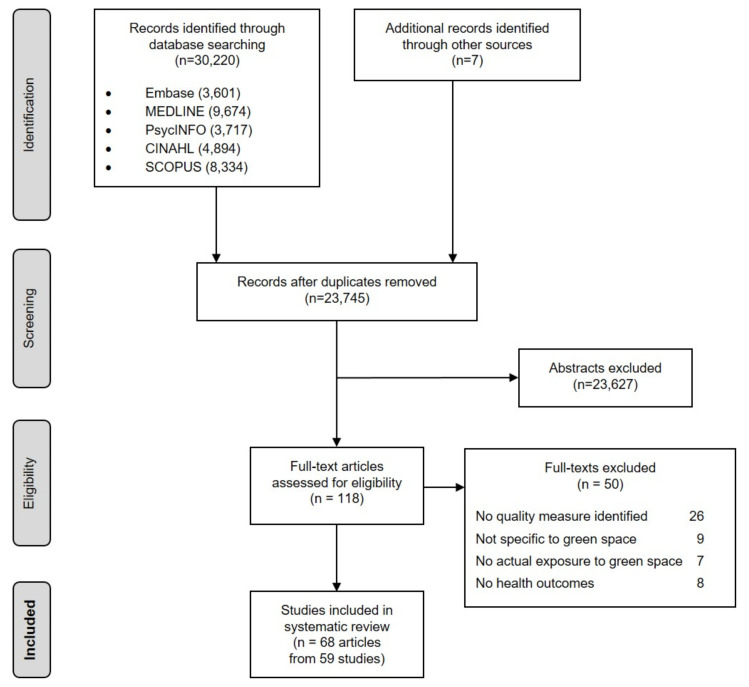
PRISMA flow diagram of study selection.

In the initial search, we identified 30,220 records, and 7 additional records were added through manual searching. After removing duplicates, 23,745 studies were included for title/abstract reviews, from which 118 full texts were selected for further screening. Fifty full texts were excluded (Supplementary File S3). The final sample comprised 68 articles from 59 studies (Figure 1). 

### 3.1. Setting and Participant Characteristics 

The 59 studies (68 articles) were conducted in 19 countries/territories and were published from January 2009 to April 2021. Most articles were based on studies conducted in the United States (US) (*n* = 17), Australia (*n* = 12) and United Kingdom (UK) (*n* = 10). The mean age of the participants ranged from 4.5 to 76.5 years. A total of 5 studies included only people aged 55 years or older [22,23,24,25,26]; 11 studies included only people under 16 years old [27,28,29,30,31,32,33,34,35,36,37]. Most studies were balanced in gender distribution, with proportions of female participants ranging from 32 to 67%. Four studies exclusively examined female participants [38,39,40,41].

Cities and inner-city neighbourhoods were the predominant settings. Seven studies took place in multi-ethnic and/or socioeconomically deprived areas [29,30,31,37,42,43,44]. One study specifically examined the differential impact of green space on children of South Asian descent versus Caucasian children [37]. The characteristics of included studies are summarised in Table 1.

### 3.2. Study Designs

**Figure 2:**
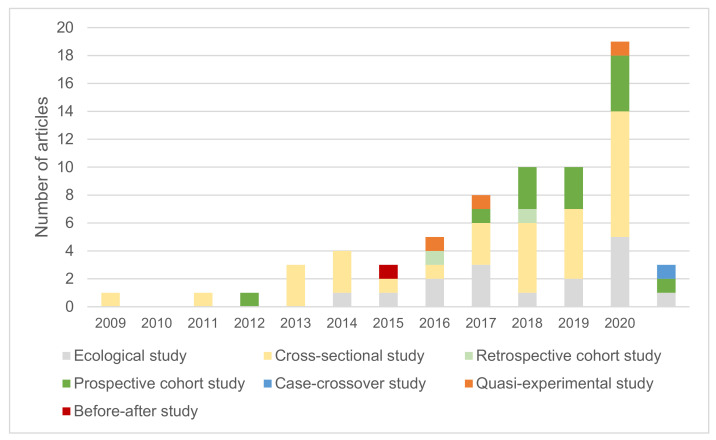
Published studies over the years, by study design.

Most included studies were cross-sectional (*n* = 32), followed by ecological studies (*n* = 16) and cohort studies (*n* = 15). Before-after (*n* = 1), quasi-experimental (*n* = 3) and case-crossover designs (*n* = 1) were rare (Table 1). The latter were relatively newer approaches published from 2015 onwards (Figure 2). All cohort studies were nested in existing longitudinal studies, usually with an additional cross-sectional survey for green space use and perceptions conducted after the initial survey waves. The follow-up time for longitudinal studies ranges from 2 to 18 years [56]. The quasi-experimental studies [55,57,87] had intervention and control groups selected in a non-random manner from two neighbourhoods with pre-determined green space qualities. The before-after study [22] was conducted among participants who participated in outdoor nature walks. The cross-over study [79] bi-directionally matched case days with the highest symptom severity scores to control days with the lowest scores, hence participants served as their own control. Among cross-sectional surveys, eight studies used convenience sampling by recruiting from park visitors [23,44,51,59,61,73,89,90]. The mean adjusted quality score among 68 articles was 0.49 ± 0.12 (scale 0–1).

### 3.3. Definition of Green Space

Most studies (*n* = 42) used a loose definition of green space to include any natural or open space, encompassing urban green space, private and community gardens, public open spaces, bushland and forest reserves, etc. Eleven studies included playgrounds and sports fields [25,35,36,37,52,53,57,60,67,71,84]. Seven studies included streetscape greenery, which referred to any vegetation cover that gave the street a green appearance [52,53,54,68,69,74,81]. Forty-seven studies used data from a geographic information system (GIS) to identify green spaces or evaluate green space characteristics. One study examined neighbourhood vegetation as viewed from within the house [22]. The most common buffer size for GIS analysis was 0.5 mile (approximately 800 m), generally aligning with a 10-min walk [82]. Detailed definition of green space in each study is outlined in Table 1.

### 3.4. Outcomes

A range of health outcomes were reported, which were classified into physical (reported by 34 studies), psychological (*n* = 25), combined physical/psychological (*n* = 10), quality of life (*n* = 5), or developmental outcomes (*n* = 3). Twenty-seven studies used objective measures of outcomes, mainly assessing physical outcomes (Table 2).

The most common tools used for physical outcomes were body mass index (BMI) (*n* = 9) [29,31,32,38,50,75,76,78,80], together with its associated anthropometric measures such as the percentage of truncal fat [27] and obesity/overweight [32,70]. Six studies investigated cardiovascular conditions such as hypertension, diabetes and coronary heart diseases [47,49,59,70,71,82]. Ten studies investigated respiratory outcomes, such as asthma and other allergic respiratory diseases [28,34,35,56,63,67,69,77,79,82]. The most common tools used for psychological outcomes were the Kessler psychological distress scale (K6-PD or K10-PD) [39,45,60] and the mental health inventory scale (MHI-5) [54,68,81]. All questionnaires used to measure psychological outcomes were self-reported by participants, indicative of the inherent subjectivity of this outcome domain. The strengths and difficulties questionnaire (SDQ) was used in studies assessing developmental outcomes. Lastly, five studies used various versions of the short form survey (SF-8, SF-12, SF-36) [23,52,54,65,81], which assess up to eight domains of health status, including physical functioning, physical role, bodily pain, general health, vitality, social functioning, emotional role, and mental health [91]. Detailed definitions of health outcomes and assessment tools are outlined in Table 3 and Supplementary File S4.

### 3.5. Green Space Qualities

Green space qualities were classified into 10 domains. Detailed definitions of green space qualities in each study is outlined in Table 3.

#### 3.5.1. Environment/Land Cover Types

There was one before-after study, seven cohort studies, one case-cross over study, seven cross-sectional studies and six ecological studies under this domain. All studies used different land cover or environment classification, commonly via adopting definitions of the data sources, some adapting [39,53,83,85] or developing their own typologies [22,86]. Detailed definitions of environment types were outlined in Table 3.

Overall, a higher land-cover diversity in the neighbourhood was protective for chronic morbidities [53] and childhood asthma [56]. Some environment types were more likely to provide health benefits than others. Vegetation patches such as grassland and tree canopy was not associated with reduced sudden unexpected deaths, but formal green spaces such as greenways and forests were [85]. People who spent time outdoor recalled greater mental restoration following visits to coastal locations and rural green space than urban green space [86]. Some environment types (“broadleaf woodland”, “arable and horticulture”, “improved grassland”, “saltwater” and “coastal” environment) were positively associated with prevalence of good health among UK citizens [83]. The observed relationship between land cover types and BMI varied across age and gender. A positive relationship with lower BMI was found with high coverage of impervious surfaces among middle-aged adults and high forest coverage among young adult males. In other age and gender groups, the relationships were non-significant [76]. More rigorous studies, however, did not report significant findings. In a before-after study, the environment type of an outdoor walk did not have significant influence on emotional states of participants [22]. A sibling matched case-control analysis of Scottish mothers and their children (1991–2010) found that infant birth weight was associated with the quantity of natural space around the mother’s home, but was unrelated to specific types of natural space (parks, woods or open waters) [41].

Similarly, the type of vegetation within green space potentially modulated its health benefits. There was consistent evidence of forests being a protective factor for obstructive airway diseases [28,34], cardiovascular diseases [49], allostatic overload [59], psychological distress and general health [45,65,74] while grassland and herbaceous vegetation were not. On the other hand, some studies showed superior benefits of shrubs and grass compared to trees in improving mental health [64] or severe allergy [79]. In low-diversity areas, certain vegetation types presented higher risks for asthma or other allergic conditions, typically non-native shrubs [56] or coniferous trees [35]. No difference in benefits between vegetation types was observed in studies of memory and dementia [46,48], depression and anxiety [26,45]. In one study, all vegetation types were shown to be protective against autism, which was potentially driven by their shared function of buffering against traffic noise and air pollution buffering [33].

#### 3.5.2. Natural Features

There was one before-after study, one cohort study, ten cross-sectional studies and three ecological studies under this domain. Natural features refer to characteristics of vegetation, animals, water bodies, and the overall naturalness of green space. Trees, flowers and fresh air [73] conferred restorative benefits to park visitors, with differential effects between genders. The higher density of trees among park vegetation was associated with lower rates of cardiovascular conditions [47,70] and a higher quality of life [24,51], but not overall general health [23]. The presence of dense shrubs, which implied lower security and safety, reduced the restorative benefits of parks [89]. Green spaces perceived as being more “natural”, such as protected areas or bushlands, provided greater benefits on mental restoration [86] and physical health [52,83]. A combination of habitat, plant, bird and insect biodiversity exhibited restorative effects, but each biodiversity component alone did not [22,42,44]. Interestingly, neither quantity or diversity of neighbourhood vegetation alone was significant predictor of stress levels, but vegetation diversity could modify the relationship between vegetation quantity and stress levels [62].

Certain green space characteristics were potentially associated with health risks. Streetscape with tree species of high allergenicity was associated with an increase in local asthma hospitalisation rates in vulnerable populations [69]. Freshwater quality was identified as an indicator of poor health status [83].

#### 3.5.3. Infrastructure and Amenities

There were nine cross-sectional studies, two quasi-experimental studies, one prospective cohort study and two ecological studies under this domain. Infrastructure and amenities refer to the availability of facilities for various purposes (recreation, resting, socialisation, etc.), the quality of paths within and leading to green space, and general maintenance. Park facilities did not reduce rates of depression [40], BMI [31,32,50,75] nor general health status of park users [23,24]. High maintenance was not associated with lower psychological distress [43] or BMI [50]. However, parks that function as recreational or sports venues may provide some cardiovascular and mental health benefits [71,84]. Mixed results were reported on the relationship between walking paths’ conditions and quality of life [25,51]. A natural experiment was conducted in disadvantaged suburbs of Melbourne, Australia, tracking psychological wellbeing of park visitors for 3 years after adding refurbishments (playground equipment, walking paths and shade) to selected parks. When compared to control parks, park refurbishments did not improve emotional states of park visitors [55]. Similarly, in the Netherlands, neighbourhoods that implemented interventions to increase accessibility and useability of green space did not see an improved general health compared to control neighbourhoods [57].

#### 3.5.4. Size

There was one prospective cohort study, six cross-sectional studies and four ecological studies and under this domain. Ten studies used spatial analysis to measure green patch size. Most studies found evidence for health benefits of larger green space for a wide range of outcomes: BMI [29,75], cardiovascular mortality [82], chronic morbidities [53], depression [42], general health status [23] and quality of life [30]. In a prospective cohort study in Perth (Australia), where residents were followed up after settling into a new neighbourhood, the increases in numbers of small parks, district parks and regional parks were each positively associated with mental wellbeing, but not the mid-sized local and neighbourhood open spaces [84]. However, some studies reported inconclusive evidence for these health benefits [24,32,78]

#### 3.5.5. Shape, Pattern and Connectivity

There were six ecological studies and two cross-sectional studies under this domain. While all studies used spatial analysis to quantify green space patterns, six studies combined health data at the spatial block level [63,67,76,77,80,82] while others conducted regression analyses using individualised data [29,30]. All studies reported positive correlation between indices measuring the shapes and distribution patterns of green patches and a wide range of outcomes, including BMI [29,76], paediatric quality of life [30], respiratory health [63,67,77] and all-cause mortality [82]. The indices include the fragmentation index (higher values indicate more fragmented green space areas), mean area of greens space (higher values indicate averagely larger green space areas), connectedness index (higher values indicate more connection between individual green spaces), aggregation/isolation index (higher values indicate more clustering of individual green spaces), shape irregularity index (higher values means more irregular shape of each green space, as opposed to round/oval shape). When stratified by gender, age and retirement status, differential benefits were observed for female and younger users [76].

#### 3.5.6. Safety

There were six cross-sectional studies under this domain. The safety of green space was associated with better quality of life [23,25,51], reduced psychological distress [43] but did not have significant effects on BMI [50] of residents. In a mediation analysis, park crimes reduced the benefits of parks on mental health [72].

#### 3.5.7. Cleanliness and Absence of Incivilities

There were three cross-sectional studies and one ecological study under this domain. Park cleanliness, either ranked by park visitors or assessed by trained auditors, was associated with lower rate of depression [42]. Evidence was inconclusive for BMI [50,78] or quality of life [24].

#### 3.5.8. Peacefulness

There were three cross-sectional studies under this domain. A lower level of “nuisance” (defined as presence of dogs, dog fouling, or young people) was not correlated with better life satisfaction nor physical health among the elderly [25]. Park users did not consider a private environment in the park important in improving their mood states [73]. On the other hand, soundscapes in parks triggered positive feelings and reduced stress [61].

#### 3.5.9. Perceived Quality/Satisfaction with Quality

There were four nested cohort studies, two cross-sectional studies, and one ecological study under this domain. In these studies, participants were asked to rank their perceived quality or aesthetics of green spaces, without a priori definition of factors to be considered. All studies examining “perceived quality” demonstrate positive association of green space’s perceived quality with health. Women living near good-quality local parks had lower rates of postpartum psychological distress or serious mental illnesses [39]. The effect on postpartum weight gain was less clear, with significant benefits only observed in areas with high vegetation coverage (≥40%) [38]. Parents’ satisfaction with green space was also linked to improved prosocial behaviour of their children [36,37]. Analysis of the Netherlands’ population data found a modest increase in life expectancy among residents living near high-quality green spaces [66]. However, perceived aesthetics of parks was neither a predictor of mood states [73] nor BMI [50].

#### 3.5.10. Combination of Features

There were one quasi-experimental study, two cohort studies, eight cross-sectional studies and two ecological studies under this domain. These studies use a mix of features from the previous domains to evaluate park quality. Detailed definitions of these composite scores were outlined in Table 3.

Five studies determined objective quality based on audits by trained assessors [32,42,54,60,68] while others asked participants to rank quality based on a set of criteria [23,25,52,58,60,68,81,87,88]. Park quality had positive benefits on reducing BMI and truncal fats in young children [27,32]. Evidence on benefits for general health were mixed [8,23,25,42,52,54,81]. Zhang et al. introduced a concept of multi-sensory experience, suggesting that visual, auditory and tactile sensation, provided by different park features, all contributed to the restorative effects of parks [88].

Three studies investigated both objectively measured and the perceived quality of green spaces, and compared their effects on health. When comparing two neighbourhoods with different socioeconomic status, the residents’ perceived quality of a green space statistically mediated the relationship between its objective quality and neighbourhood satisfaction, but did not have any direct effect of wellbeing [87]. Only objective quality reduced psychosocial distress (K6-PDS questionnaire) in one study [60] while only perceived quality improved mental wellbeing (MHI-5 questionnaire) in another study [68].

## 4. Discussion

Overall, our review demonstrates evidence of health benefits associated with a wide range of green space qualities. Increasing research interest in green space qualities was demonstrated (Figure 2) and this aligns with rising interest in urban greening to counter the health and climate impacts of urbanisation [6]. The COVID-19 pandemic may also have amplified attention on this topic from academics and policymakers, as communities in many countries have flocked to green spaces as a means of coping with lockdowns and socioeconomic disruption [92,93]. After excluding results with a study quality assessment score under 50 (*N* = 32), evidence showed consistent positive associations with health with the green space qualities we classified as “environment types”, “natural features”, “shape and connectivity”, and “objective quality scores”. Limited evidence was found on the health benefits of improving infrastructure or amenities in green spaces. Research gaps were identified for the following green space qualities: peacefulness, safety and absence of incivilities; needs-specific or culture-appropriate amenities, and soundscape characteristics.

### 4.1. Green Space Qualities

The most commonly assessed qualities of green spaces were the environment types of the natural space, as well as vegetation types and other natural characteristics. Our review shows that some environment types were linked to positive health outcomes more than others [41,83,86]. Health benefits were observed when the environment type facilitated age- and gender-appropriate physical activities. For example, middle-aged adults group preferred built facilities with paved paths for exercising whereas young adults prefer forested areas with unobstructed grounds for athletic, adventurous activities such as hiking, trail-running or mountain biking [76,82]. Therefore, preserving diversity in land cover types (e.g., structured versus natural) may be a potential option to enhance health benefits of green spaces, especially in dense urban areas with limited options for expansion. Moreover, green space designs might be optimized for health through tailoring to local community profiles, to bring people together and to enable them to do what they find nourishing. This requires consultation and it is likely that certain qualities may be a source of conflicting views. For example, accommodating for birds in green spaces may be viewed positively for their provision of restorative soundscapes and an enhanced feeling of connectedness with nature, but also negatively due to the timing of their sounds, impacts on property (e.g., droppings) and occasional swooping that may create a lack of felt safety [94].

Evidence indicated that some vegetation types may be more beneficial towards particular health outcomes than others. Tree canopy and forests were more consistently associated with better cardiovascular and respiratory health than grassland [47,59,67,79]. A reason may be that trees permit and promote restoration while also providing shade that helps to activate walking and active transportation (particularly in hot climates), whereas grass and shrubs might not convey the same range and levels of benefit [76]. Moreover, because of their foliage, evidence indicates that forests have the capacity to intercept airborne pollutants and buffer against traffic noise, alleviating oxidative stress and reducing risks of atherosclerotic diseases [95]. On the other hand, shrubs may impede visibility and reduce levels of felt safety, while large areas of open grass may reduce walkability (especially if it is walled or fenced-off, as can be the case for private green spaces like golf courses) [74,76,89]. Importantly, this may reflect an interaction between vegetation type and other contextual factors, such as levels of crime, nearby land-use and transport infrastructure. Further research that examines potential contingencies of association between vegetation types and health outcomes within the context of other land-uses is warranted.

Interestingly, many studies in the facilities/amenities domain show no statistically significant associations with physical or mental health, despite evidence that some of these qualities are associated with physical activity [90]. This might be because different types of facilities may result in different forms of behaviour, some of which may instead promote sedentary forms of leisure (e.g., seating) or detract from the perceived ‘naturalness’ appealed by certain park users (e.g., some sports facilities that use synthetic materials) [96]. Moreover, some studies may log the availability facilities but not their condition and usability. For instance, access to areas of parks and particular buildings may be difficult for people with functional limitations, while there may also be cultural or social factors that influence whether a particular facility is considered accessible [97].

Some qualities have a small evidence base, such as safety, tranquility or absence of incivilities. Most of this evidence focused on psychological wellbeing or quality of life using Likert-type rankings or the number of unwell days. Future studies on these aspects will benefit from using robust, validated questionnaires featured in other quality domains, such as MHI-5, PRS or PANAS [22,55,68,89]. Moreover, perceived safety of public spaces can be influenced by neighbourhood characteristics and social vulnerabilities, which need to be accounted for in future studies.

Some quality domains were not featured among the included studies. Availability of needs-specific amenities, such as for people living with particular disabilities, may encourage more inclusive park usage and increase the potential to reduce health inequity [3,98]. Tailoring park amenities and features to the local communities, such as instructions in multiple languages, accommodation (and celebration) of cultural traditions and rituals, etc., may be particularly important in multi-cultural neighborhoods [99,100]. One study examined the feelings evoked by soundscape [61], but the constituents of soundscape that provide therapeutic effects, such as sounds of nature, human activities or traffic noise, were not elucidated. Types of bird songs were previously studied, but as sound clips rather than actual exposure inside parks [89].

### 4.2. Health Outcomes

Physical health is the most commonly assessed set of health outcomes. Most studies showed evidence of potential benefits for anthropometric measures (BMI and obesity) and respiratory health (allergic diseases). Understandably, there are established frameworks explaining how green spaces reduce obesity via promoting physical activities [96,101] and protects against respiratory diseases via regulating temperature and air pollution [77]. Only 7 out of 34 studies on physical health examined associations with cardiovascular diseases.

Based on existing evidence, higher quality green space may reduce cardiovascular mortality and incidence of cardiometabolic diseases [47,70,71]. However, evidence for associations with specific cardiovascular diseases was small. Consistent evidence from this review indicated a range of probable mental health benefits linked with various green space qualities. This aligns with existing conceptual frameworks, which suggest green spaces can confer mental health benefits via reducing stressor exposures and replenishing mental resources for coping [3].

Although the evidence base is substantial for physical and psychological health outcomes, there is granularity in the quality of outcome measurement tools. For physical health, a number of studies relied on general health questionnaires such as SF-12 and SF-36. These have a low administrative burden and good internal validity [91], but responses may differ among age, education or ethnicity subgroups [102], which may explain conflicting findings among these studies. For mental health, some studies used self-ranked Likert-type questions, which lacked reliability and consistency compared to validated questionnaires like the MHI-5, K6-PDS or CED-S. A potential approach for future studies is to use quantifiable biological measure to validate subjective questionnaires, such as hair cortisol levels as a proxy for stress [62].

Few studies investigated child development. This could be the focus of future studies, as evidence suggests possible health benefits linked to reduced maternal stress during pregnancy [33] and opportunities for play and socialisation during time spent in green spaces [36,37].

Certain outcomes were not featured in the included studies. Vegetation types and structure influence their ability to regulate pollution and local climate, and thus will have differential effects on heat-related health risks [103]. Postpartum distress was examined [39], but the effects on antenatal depression or neonatal outcomes were not investigated. This is an important topic, as the greenness of the environment was associated with reduced risks of low birth weight and preterm delivery [104].

### 4.3. Quality of Study Designs

Overall, the level of evidence certainty for health benefits of green space quality remains low.

This is due to two important reasons. Firstly, there was a high degree of heterogeneity in study designs, green space and green space quality definitions, and outcome measurements. Some studies use factor analysis to derive the qualities, which make it difficult to find out the definitions behind the derived terms, especially when the survey questionnaires were not included [40,52]. Many studies ask participants to rank certain qualities or report health outcomes on a Likert scale-type questions, without defining the quality being surveyed for the participants. These potentially introduce bias in response and are a major limitation among studies in this topic. Even with GIS methods, which are deemed more reliable and reproducible in quantifying green space exposure, variations in proximity radius and buffer zones make it difficult to compare results across studies.

Secondly, none of the included studies were randomised trials, which resulted in a lower overall quality of evidence.

Only 6/10 domains featured evidence from longitudinal cohort studies or interventional studies (before-after and quasi-experimental studies), namely the domains of environment types, natural features, infrastructures and amenities, size, perceived quality, and combination of features. Within each domain, cross-sectional and ecological studies often accounted for more than half of the evidence base. The prevalence of observational studies is characteristic of environmental health research, which faces intrinsic logistical and ethical challenges in designing rigorously controlled trials [105]. Nonetheless, observational studies have their limitations [106]. Cross-sectional surveys do not permit inference of causation. In our review, many cross-sectional surveys used convenience sampling, which could introduce selection bias due to seasonal weather, site of surveys or time of day. Longitudinal studies can factor in temporal relationship between green space exposure and health outcomes. They also enabled adjustment for factors that can influence health outcomes, such as demographic characteristics, measures of poverty and deprivation, and socioeconomic status (income, education and employment) (Table 1). However, many cohort studies in our review were nested in longitudinal health surveys that did not routinely collect data on green space quality, and only achieved so via a cross-sectional survey or geospatial analysis [36,38,39], again making it impossible to establish temporal causation. Although ecological studies echo the principles of environmental health policies, their generalizability is limited. By assuming that green space exposure applies uniformly to all individuals within a census tract or administrative area, these studies do not control for individual health and preference, and thus may lead to incorrect inferences (“ecological fallacy”). The use of multiple databases in GIS analysis, featured in many of our studies, also raises the possibility of spatial autocorrelation and mismatched data sources, etc. [34,83]. Before-after studies and quasi-experiments are pragmatic designs that support causal inference by establishing a clear temporal relationship between exposure and outcomes and controlling for confounding factors. They provide real world effectiveness of complex interventions, and are thus compatible with population policies [107].

It is important to note that, although longitudinal cohort studies and interventional studies were less prevalent, they have methodological strengths that cross-sectional and ecological studies do not. In our review, limiting analysis to these studies did not change the overall conclusion across all quality domains.

### 4.4. Future Directions

Innovative trial designs have been featured in this review, namely quasi-experimental studies using controlled parks or neighbourhoods [55,57]. In addition, controlled intervention design had been used in forest therapy trials, which allowed for robust pre-post measurements of cardiovascular outcomes such as blood pressure, heart rate and oxygen saturation [108]. However, high logistical demands often limited the duration of these trials and precluded studies of long-term (child development) or high-risk outcomes (childbirth, cardiovascular events). Studies nested in cohort follow-up studies [28,36,37,41,49,60,84] are a promising approach by leveraging on well-designed longitudinal studies with annual follow ups, comprehensive baseline data collection, and large sample sizes for robust statistical power. Where randomisation is not possible, study data could be analysed using interrupted times-series analysis, which adjusts for some effects of context and individual health variations over time [69].

Satellite imagery and GIS should still be part of the essential toolbox for green space quality studies, as long as GIS data is linked to patient-level data instead of being aggregated at ecological unit levels. GIS has proven useful in combining cartographical datasets, identifying and classifying land cover types. Recent advances in geospatial big data also introduced new approaches to assessing green space exposure, such as eye-level exposure (street view imagery) as opposed to overhead exposure (satellite imagery) [109]. In addition, GIS technology has enabled new indices for quantifying green space size, shape and connectivity [30,82]. By virtue of defined formulae, these indices were reproducible and reliable, and could be used in various statistical analyses.

Our findings showed that perceived green space quality, even without any judging criteria, can predict health benefits [36,37,39,66]. This is an important consideration, given that spatial environmental indicators (size, greenness, aesthetics) do not always corresponded with user perceptions [110]. Therefore, it is advisable for future studies to measure both perceived and objective quality when assessing health benefits. This approach has the dual benefits of ensuring internal validity of the subjective quality measurement, while accounting for any mediating effect of user perceptions on the objective quality [60,68].

Several studies used a composite quality score that aggregated across several domains (e.g., Public Open Space Tool). Although a composite score approach can reflect the complexity of green space quality, coverage can be restricted to attributes related to facilities, safety and cleanliness, which are shown in our review to have little association with health so far. RECITAL, the latest quality assessment index developed to address this gap, incorporates other quality domains such as suitability for activities, land cover types and biodiversity [111], which generally aligns with our classification. This index can be stratified into single-item or sub-section scores, allowing researchers to investigate specific aspects of quality, which is a shortcoming commonly associated with aggregated scales. Comprehensive indices such as this should be explored in future studies. Last but not least, there is a need for a new index that aggregates qualities across networks of multiple green spaces of various shapes and attributes. This may be particularly salient within higher density contexts, where multiple smaller green spaces exist with each containing a small number of qualities, but larger ones that may incorporate many more qualities do not.

### 4.5. Strengths and Limitations

The strength of our review is its breadth of coverage, as we formulated our search strategy intentionally to capture across a range of health outcomes, potential qualities and green space types. Our review is the first to capture the diverse evidence conducted in this area and map them into domains of quality. Nonetheless, our review was not without limitations. As the concept of green space quality was not well-defined, we took a holistic approach but our review could still potentially miss out relevant studies that did not use conventional descriptors of quality. Our review only included studies written in English, and in view of more emerging research on park designs from China in recent years [112], publication bias due to exclusion of non-English articles was possible. Although our review was structured based on established protocols, the screening process was subjected to some degree of subjectivity due to a lack of standardized definitions in this topic.

## 5. Conclusions

Research on green space quality and health has increased in volume, especially since 2016. A high degree of heterogeneity was observed in study design, and the definitions of quality and outcomes measured. Environment types, vegetation types, and the size and connectivity of green spaces, were associated with physical and mental health outcomes, with differences by age and gender. The associations indicative of health benefits were more consistent in populations with more tree canopy, but not more grassland. Qualities such as safety, cleanliness and aesthetics tended to be investigated with weaker study designs. Both objective and subjective quality demonstrated positive effects on health outcomes. There is a need for more experimental studies or well-designed prospective studies that incorporate longitudinal measures of green space qualities and outcome-appropriate confounders. Green space indices should account for form, pattern, networks, and both objective and perceived qualities.

## Figures and Tables

**Table 1:** Study characteristics.

Study	Location *	Study Design	Sample Size **	Population	% Female	Age	Description of Green Space Types	Mediating Factors	Factors Adjusted in Analysis
Aerts, 2020 [34]	Belgium	Eco	1872 census tracts	Children aged 6–12 and 13–18 years	N/A ^≠^	Range: 6–18	Grassland (permanent grassland, hay meadows and lawns); gardens (ornamental gardens and vegetable gardens); forest (coniferous, mixed and broadleaved woodlands)	-	Time, green space coverage, mean annual PM10 concentration, %houses with basic or insufficient, administrative region
Astell-Burt, 2019 [45]	New South Wales, Australia	CS-Pros	46,786	Adults ≥ 45 years old	53.8	Mean: 61.0 ± 10.2	Tree canopy, grass and other low-lying vegetation	-	Age, sex, household income, employment status, education, couple status
Astell-Burt, 2020 [46]	New South Wales, Australia	CS-Pros	109,688	Adults ≥ 45 years old	52.3	Median range: 55–64	Trees and grass	-	Age, gender, economic status, education, household income, couple status, area-level disadvantage, total green space
Astell-Burt, 2020 [47]	New South Wales, Australia	CS-Pros	46,786	Adults ≥ 45 years old	53.8	Median range: 55–64	Street trees and trees in parks	-	Age, gender, couple status, education, household income, employment
Astell-Burt, 2020 [48]	New South Wales, Australia	CS-Pros	45,644	Adults ≥ 45 years old	N/R	N/R	Tree canopy, open grass and shrubs	-	Age, sex, living arrangement, education, household income, economic status
Astell-Burt, 2021 [49]	New South Wales, Australia	CS-Pros	45,644	Adults ≥ 45 years old with type 2 diabetes mellitus	N/R	N/R	Tree canopy, open grass	-	Age, sex, living arrangement, education, household income, economic status
Bai, 2013 [50]	Kansas, USA	CSS	893	Urban residents living within 0.5 miles from parks	60.7	Mean: 50.9 ± 16.5	Parks	-	Age, sex, race/ethnicity, income, past park use
Bird, 2016 [27]	Canada	CS-Retro	380	Caucasian children 8–10 yo with at least one obese parent	52.4	Mean: 9.7 ± 0.89	Parks and open spaces	-	Age, sex, puberty, household income
Bojorquez, 2018 [40]	Tijuana, Mexico	CSS	2345	Urban female residents	100.0	Mean: 37.0	Parks	Being active in a public space	Age, marital status, children, SES (employment, education), park coverage
Camargo, 2017 [51]	Bucaramanga, Colombia	CSS	1392	Urban park visitors	58.4	Median: 42 (28–55)	Zonal and local urban parks	-	Education, health status, walking difficulty, anxiety/depression, visiting with a companion, active park use
Carter, 2014 [52]	Perth, Australia	CSS	440	Residents in inner-city and suburban neighbourhoods	64.0	Range: 45–54	Parks, gardens, play and social green spaces, bushland, sports fields, streetscapes, private yards	-	Age, SES (income, education), family structure, living arrangement, neighbourhood
Dennis, 2020 [53]	Manchester, UK	Eco	1673 LSOAs	Urban residents in young vs. old neighbourhoods of various income levels	N/R	Old areas: >23.6% population are ≥60 yo Young areas: ≤23.6%	Public parks, recreational spaces (playing fields, allotments and sports facilities), landscaped open spaces, private gardens, institutional land, previously-developed land, peri-urban and informal urban greenery (street trees, road verges)	-	Age, sex, income, employment, barriers to housing and services, educations/skills/training, crime levels
Dillen, 2012 [54]	Netherlands	CSS	1553	General population	52.0	Median range: 45–65	Streetscape greenery Green areas: parks, forests, nature and recreation areas	-	Age, sex, SES (education, income)
Dobbinson, 2020 [55]	Melbourne, Australia	QES	1670	Park visitors in a deprived neighbourhood	44.7	Median range: 34–37	Parks	-	-
Donovan, 2018 [56]	New Zealand	CS-Retro	39,108	Adults aged 18	48.7	Mean: 18.0 ± 0	Urban parkland/open space, grassland, herbfield, orchards, vineyards, crops, grassland, freshwater and saline vegetation, flaxland, gorse, shrublands, mangroves, forests and hardwoods	-	Premature birth, low birth weight, antibiotic use, parental smoking, ethnicity, birth order and number of siblings, and parental occupation
Droomers, 2015 [57]	Netherlands	QES	48,132	Residents living in neighbourhoods with history of green intervention projects	N/R	N/R	Parks, natural playgrounds, community gardens or fruit orchards, children’s farms, fishponds, public allotment gardens, etc.	-	Living circumstances, neighbourhood, characteristics, safety
Dzhambov, 2018 [58]	Plovdiv, Bulgaria	CSS	399	High school and university students 15–25 yo	32.0	Mean: 17.89 ± 2.27	Any green space	Restorative quality, social cohesion, physical activity, noise annoyance, perceived air pollution	Age, sex, ethnicity individual-level SES, time spent at home/day, duration of residence, population density, month
Egorov, 2020 [59]	North Carolina, USA	CSS	186	Urban residents	67.2	Mean: 37.1	Trees and forest, grass and other herbaceous	-	Age, smoking status, education, BMI, density of residential units, concentration of NOx from local traffic, geographic coordinates
Feng, 2018 [39]	Australia	CS-Pros	3897	Mothers in postpartum period	100.0	Median range: 35–39	Parkland	-	ARIA score, SEIFA score
Feng, 2019 [38]	Australia	CS-Pros	3843	Mothers in postpartum period	100.0	Median range: 40–44	Parkland	-	Maternal age, SES (education, employment), years since childbirth, indigenous status, area disadvantage, remoteness (seia & aria), family structure
Francis, 2012 [60]	Perth, Australia	CS-Pros	911	Residents moving to newly-built homes	62.0	Median range: 40–59	Public open spaces: parks, recreational grounds, sports fields, commons, esplanades and bushland/wilderness	-	Age, sex, SES (income, employment, education), marital status, children living at home, neighbourhood SES
Gernes, 2019 [28]	Ohio & Kentucky, USA	CS-Pros	478	Children aged 7 years	Cases: 42.4 Control: 49.0	Mean: 7.0 ± 0	Trees and grass	-	Race, sex, environmental tobacco smoke exposure, exposure to traffic-related air pollution, mother’s education, neighbourhood SESneighbourhood SES (7 years).
Herranz-Pascual, 2019 [61]	Vitoria-Gasteiz, Spain	CSS	137	Urban park visitors	54.0	Mean: 42.3 ± 14.2	Urban parks	-	Age, sex, education, acoustic and environmental comfort of the environment (13 dimensions in a semantic differential scale)
Honold, 2016 [62]	Berlin, Germany	CSS	32	Residents living in inner-city neighbourhoods	59.4	Mean: 36.0 ± 10.2	View of vegetation from windows	-	Age, exercise, range of view, perceived chronic stress
Jaafari, 2020 [63]	Tehran, Iran	Eco	87 hexagons	General population	N/R	N/R	Green space	Air pollution	-
Jarvis, 2020 [64]	Vancouver, Canada	CSS	1,960,575	General population	51.7	Median range: 25–44	Coniferous trees, deciduous trees, shrubs and grass-herbs	-	Age, sex, racial/cultural background, education level, household income, persons < 18 years old in household, urbanicity
Jiang, 2020 [65]	USA	Cross-sectional study	212	General population	57.1	Median range: 30–45	Tree canopy, low-level vegetation	-	Age, income
Jonker, 2014 [66]	Netherlands	Eco	1190 neighbourhoods	General population	N/R	N/R	Any green space except horticulture and streetscape vegetation	-	Sex, neighbourhood income, household disposable income, nursing home migration of frail elderly
Kim, 2014 [29]	Texas, USA	CSS	61	Primary school students 9–11 yo from a deprived area with large Hispanic population	60.7	Mean: 10.1 ± 0.67	Tree canopy	-	Sex, maternal marital status and education, number of cars, neighbourhood satisfaction, accessibility to play areas
Kim, 2016 [30]	Texas, USA	CSS	92	Primary school students 9–11 yo from a deprived area with large Hispanic population	62.0	Mean: 10.0 ± 0.68	Tree canopy	-	Age, sex, maternal employment status, physical activity time, TV watching hours, neighbourhood environmental perceptions
Kim, 2021 [67]	Los Angeles, USA	Eco	2301 census tracts	General population	N/R	N/R	Private green spaces (yards, gardens, landscaped areas), semi-public green spaces (golf courses, schools, cemeteries, agricultural lands), public green spaces (parks and recreational areas)	-	Poverty rate, education, ethnic group, children population, senior population
Kruize, 2020 [68]	Europe	CSS	3947	Urban residents	55.4	Mean: 51.4 ± 16.0	Natural outdoor environment: any outdoor spaces that contain green or blue natural elements (street trees, forests, city parks, water bodies)	-	Age, sex, education, ndvi within 300 m, city
Lai, 2019 [69]	New York City, USA	Eco	174 zip codes	General population	N/R	N/R	Street trees	-	Buffering traffic noise and air pollution
Leng, 2020 [70]	Harbin, China	CSS	4155	Urban residents of a winter city	47.7	Mean: 54.6 ± 10.3	Any green space	-	Age, sex, SES (education), smoking, cardiovascular family history
Marselle, 2015 [22]	UK	BAS	127	Elderly ≥ 55 years who participated in outdoor walks	55.5	Range: 55–74	Natural and semi-natural places, green corridors, urban green spaces, farmland, urban public spaces, coastal spaces	Perceived restorativeness	Type of environments, walk characteristics (duration, intensity)
McCarthy, 2017 [31]	USA	CSS	13,469	Children in elementary schools in a multi-ethnic, deprived region	49.2	Mean: 9.7 ± 0.99	Parks	-	Age, sex, race/ethnicity, SES (education, income), nativity, marital status, children in household, self-reported health
McEachan, 2018 [37]	UK	CS-Pros	805	Children of age 4 of South Asian parents in a multi-ethnic, deprived city	50.0	Mean: 4.5 ± 0.4	Public parks, play areas for children, sports fields, any natural habitats with plants and vegetation	-	Demographics, SES, maternal health behaviours, maternal mental wellbeing
Mears, 2020 [32]	Sheffield, UK	Eco	345 LSOAs	Children in first and final years of primary school	N/R	Range: 4–5 and 10–11	Any natural land covers, including water	-	Age, sex, income deprivation, air pollution, address density
Mears, 2020 [42]	Sheffield, UK	Eco	345 LSOAs	General population in a highly-deprived region	N/R	N/R	Any natural land covers, including water	-	Age, sex, income deprivation, air pollution, smoking rates, address density
Ngom, 2016 [71]	Montreal and Quebec, Canada	Eco	N/A	General population	N/R	N/R	Parks and woodlands, golf courses or any sport facilities	-	Age, ambient air pollution, immigrant population, total population, social and material deprivation scores
Nishigaki, 2020 [26]	Japan	CSS	126,878	Elderly ≥ 60 years with pollen allergy	51.5	Median range: 70–74	Fields (rice paddy, crops), grassland, trees (deciduous, evergreen)	-	Age, sex, education, household income, living with others, employment, frequency of going outside, driving a car, residence duration, total daylight, annual snowfall amount, annual rainfall, residential population density
Orstad, 2020 [72]	New York City, USA	CSS	3652	Urban residents in areas with high prevalence of obesity	58.9	Median range: 45–64	Parks	Park use for physical activity, park crime	Age, sex, race/ethnicity, language of interview, SES (education, income, employment, car ownership), marital status, BMI, perceived traffic volume, perceived retail access, survey wave and strata
Parmes, 2020 [35]	Europe	CSS	8063	Children aged 3–14 years	47.7	Range: 3–14	Green urban areas, sport and leisure facilities, broad-leaved forest, coniferous forest, mixed forest, natural grassland, moors and heathland, sclerophyllous vegetation, transitional woodland/shrub	-	Age, sex, BMI, parental history of allergy, maternal education, parental smoking
Pazhouhanfar, 2018 [73]	Gorgan, Iran	CSS	250	Urban park visitors	57.3	N/R	Parks	-	sex
Pope, 2018 [43]	Sandwell, UK	CSS	578	Urban residents in a deprived area	51.1	Median range: 40–59	Any green space	-	Age, sex, index of multiple deprivation
Putra, 2020 [36]	Australia	CS-Pros	4969	Children 4–15 yo	48.7	Range: 4–15	Parks, playground and place space	-	Age, sex, ethnicity (indigenous), non-English speaking, family SES, family structure, SEIFA score, ARIA score, neighbourhood safety
Reid, 2017 [74]	New York City, USA	CSS	1387	Urban residents	63.6	Mean: 44.7 (Range 18–90)	Streetscape greenery	-	Age, sex, race/ethnicity, season, neighbourhood tenure, individual SES (income, education), area-level SES (% living below poverty, % unemployed), no2, % park and non-park open space
Richardson, 2018 [41]	Scotland	CS-Pros	46,093	Mothers	100.0	Median range: 25–29	Natural space: all public and private natural surfaces (vegetation, water, sand, mud and rock)	-	Infant’s sex, parity, gestational age, year of birth, season of conception, maternal age, height, education, ethnicity, tenure, smoking during pregnancy
Rundle, 2013 [75]	New York City, USA	CSS	13,102	Urban residents	64.0	Median: 45	Parks	-	Age, sex, race/ethnicity, individual SES: education, neighbourhood SES: % residents in poverty, %black/African American, %Latino/Hispanic, % park land by park size
Sander, 2017 [76]	Ohio, USA	Eco	546 census blocks	General population	N/R	Mean: 43.02 ± 4.37	Publicly accessible conservation lands, recreational parks and cemeteries	-	Age, ethnicity, education, urban development intensity, population density, household income
Shen, 2017 [77]	Taipei, Taiwan	Eco	48 districts	Urban residents	N/R	N/R	Green structure	Temperature, primaryand secondary air pollutants	-
Stark, 2014 [78]	New York City, USA	CSS	44,282	Urban residents	58.5	Mean: 26.6 ± 5.5	Parks	-	Age, sex, race/ethnicity, SES (education, income), nativity, marital status, children in household, self-reported health
Stas, 2021 [79]	Belgium	CCS	189	Adults ≥ 20 years old with pollen allergy	59.3	Mean: 40.4 ± 9.9	Gardens, grassland and forests	-	Age, sex, exposure to birch pollen and air pollutants, geographic regions
Sugiyama, 2009 [25]	UK	CSS	271	Elderly ≥ 65 yo	60.0	Mean: 75 ± 7.2	Neighbourhood open spaces: parks, community gardens, play and sports areas, village greens, river or canal banks, beaches	-	Age, functional capability, education
Tan, 2019 [23]	Tainan, Taiwan; Hong Kong	CSS	326	Elderly ≥ 55 yo	56.0	Median range: 70–79	Urban green space	-	Age, park usage
Tsai, 2016 [80]	USA	Eco	52 MSAs	General population	N/R	N/R	Forests (woody vegetation > 6 m in heights, including deciduous, evergreen and mixed); shrubland (woody vegetation and young trees < 6 m in heights); herbaceous (grassland, wildflowers)	-	Total population, total housing units, household income, % African American population
Vries, 2013 [81]	Netherlands	CSS	1641	General population	51.0	Mean: 51.0 ± 16.0	Any visible streetscape vegetation: flower boxes, green facades, view of woodlands, etc.	Stress, social cohesion, green activity	Age, sex, SES (education, income), life events, children, smokers, excessive drinkers
Wang, 2019 [82]	Philadelphia, USA	Eco	369 census tracts	General population	53.4	N/R	Tree canopy, grass cover and shrub cover with area ≥ 83.6 m^2^	-	Age, sex, ethnicity, education, population density, land area
Wheeler, 2015 [83]	UK	Eco	31,672 LSOAs	General population	N/R	N/R	Any natural landscape	-	Age, sex, SES (income, education and employment), urban/rural status, indices of deprivation
Wood, 2017 [84]	Perth, Australia	CS-Pros	492	Residents moving to newly-built homes	61.6	Mean: 47.8 ± 12.1	Parks, gardens, reserves, grassed open spaces and any freely-accessible sports fields	-	Age, sex, SES (income, employment, education), marital status, children living at home
Wood, 2018 [44]	Bradford, UK	CSS	128	Urban park visitors in a multicultural, deprived area	46.0	Median range: 36–45	Formal parks and recreation grounds	-	Age, sex, ethnicity, connected to nature
Wu, 2017 [33]	California, USA	Eco	543 districts	Children in public elementary schools	49.1	Range: 5–12	Forest, grassland, tree canopy	-	Sex, household income, race
Wu, 2018 [85]	North Carolina, USA	Eco	187 census tracts	General population	N/R	N/R	Forest, grassland, tree canopy, and greenway	-	Age group, population density, household income, %Asian population
Wyles, 2019 [86]	UK	CSS	4515	General population	52.2	Median range: 35–44	Any open space: parks and canals in cities and towns; coast and beaches; farmland, woodland, hills and rivers in the countryside	Connectedness to nature	Age, sex, SES, activities taken during visit, average time spent, distance to site, mode of transport, presence of companions
Zhang, 2017 [87]	Netherlands	QES	223	Residents from two neighbourhoods with contrasting green space qualities	55–61	Mean: 49.6 years (exposure) 39 years (comparison)	Any green space	Neighbourhood satisfaction	Quantity of green space, age, length of residence, income
Zhang, 2019 [88]	Guangzhou, China	CSS	250	Urban park visitors	58.0	Median range: 31–45	Park with a flowers garden, an entertainment and leisure zone, an elderly activity area, a forest rest zone, and a logistics management zone	Emotional responses, behavioural activities in parks	-
Zhang, 2019 [24]	Hong Kong	CSS	909	Residents ≥ 65 years from elderly health centres and community centres	66.3	Mean: 76.5 ± 6.0	Parks	-	Age, sex, education, area-level SES, marital status, living arrangement, housing type, household with car, type of recruitment centre, number of current health problems
Zhu, 2020 [89]	Harbin, China	CSS	240	Urban park visitors	43.0	Median range: 20–29	Island/archipelago within a city	-	-

* Abbreviations: CCS: case-crossover study; CSS: cross-sectional study; CS-Retro: retrospective cohort study; CS-Pros: prospective cohort study; QES: quasi-experimental study; BAS: before-after study; Eco: ecological study; ** Default unit is person unless specified otherwise. Abbreviations: DA: dissemination area; LSOA: lower layer super output area; MSA: metropolitan statistical area. ^≠^ analysis was stratified by sex.

**Table 2:** Mapping of measures used for assessment of green space qualities and outcomes.

Green Space Quality Domain	All Studies	Studies Using Objective Measures to Assess Green Space Quality	Psychological Outcomes			Physical Outcomes			Combined Physical-Psychological Outcomes			Developmental Outcomes			Quality of Life Outcomes		
			Both Subjective and Objective Measure *		Objective Measure Only	Both Subjective and Objective Measure *		Objective Measure Only	Both Subjective and Objective Measure *		Objective Measure Only	Both Subjective and Objective Measure *		Objective Measure Only	Both Subjective and Objective Measure *		Objective Measure Only
Environment/land cover type	22	20	8	(36.4%)	1	12	(54.5%)	8	4	(18.2%)	0	1	(4.5%)	1	0	(0.0%)	-
Natural features	15	11	5	(33.3%)	0	5	(33.3%)	4	3	(20.0%)	0	0	(0.0%)	-	2	(13.3%)	0
Infrastructure and amenities	14	10	4	(28.6%)	0	6	(42.9%)	4	2	(14.3%)	0	0	(0.0%)	-	3	(21.4%)	0
Size	11	11	2	(18.2%)	1	6	(54.5%)	5	2	(18.2%)	1	0	(0.0%)	-	2	(18.2%)	1
Shape, pattern and connectivity	8	8	0	(0.0%)	-	7	(87.5%)	5	0	(0.0%)	-	0	(0.0%)	-	1	(12.5%)	1
Safety	6	0	2	(33.3%)	0	1	(16.7%)	0	2	(33.3%)	0	0	(0.0%)	-	2	(33.3%)	0
Cleanliness and absence of incivilities	5	4	1	(20.0%)	1	3	(60.0%)	1	0	(0.0%)	-	0	(0.0%)	-	1	(20.0%)	0
Peacefulness	3	0	2	(66.7%)	0	0	(0.0%)	-	1	(33.3%)	0	0	(0.0%)	-	1	(33.3%)	0
Perceived quality/ Satisfaction with quality	7	0	2	(28.6%)	0	3	(42.9%)	1	0	(0.0%)	-	2	(28.6%)	0	0	(0.0%)	-
Combination of features	13	6	6	(46.2%)	0	5	(38.5%)	2	5	(38.5%)	0	0	(0.0%)	-	2	(15.4%)	0

* Expressed as a percentage of all studies under respective green space quality domain.

**Table 3:** Summary of findings.

Study *	Measure of Quality	Tool(s) Used to Assess Green Space Quality **	Outcome	Outcome Assessment Tool **	Direction of Effect ^≠^
Environment/land cover type (*n* = 22)					
Marselle, 2015 (*n* = 127) [22]	Environment types: natural and semi-natural places, green corridor, urban green space, farmland, urban public spaces, coastal, mixture	Self-reported	Positive & negative affect	PANAS scale	(o)
Stas, 2021 (*n* = 189) [79]	Vegetation species and cover types: trees vs. grass	GIS analysis	Severe tree pollen allergy event	Self-reported	(+)
Astell-Burt, 2020 (*n* = 109,688) [46]	Vegetation cover types: trees vs. grass	GIS analysis	Dementia: first medication prescription, first hospitalisation and deaths	Medical records	(+)
Astell-Burt, 2019 (*n* = 46,786) [45]	Vegetation cover types: trees, grass vs. low-lying vegetation	GIS analysis	Psychological stress; depression/anxiety; general health	K10-PDS; self-reported	(+)
Richardson, 2018 (*n* = 46,093) [41]	Natural space types: parks, woods, open waters	GIS analysis	Live births	Medical records	(+)
Astell-Burt, 2020 (*n* = 45,644) [48]	Vegetation cover types: trees vs. grass	GIS analysis	Memory complaints; self-rated memory	Semantic differential scale	(o)
Astell-Burt, 2021 (*n* = 45,644)	Vegetation cover types: trees vs. open grass	GIS analysis	CVD mortality, CVD events, AMI	Medical records	(o)
Gernes, 2019 (*n* = 478) [28]	Land cover diversity	GIS analysis	Outdoor allergen sensitisation; allergic rhinitis	Skin prick tests; clinically diagnosed	(–)
Donovan, 2018 (*n* = 39,108) [56]	Vegetation cover types	GIS analysis	Childhood asthma	Medical records	(+)(–)
Parmes, 2020 (*n* = 8063) [35]	Forest types: deciduous, coniferous vs. mixed	GIS analysis	Wheezing, asthma, allergic rhinitis, eczema	Parental reported	(–)
Jarvis, 2020 (*n* = 1,960,575) [64]	Land cover types: coniferous, deciduous, shrub, grass-herbs, water, buildings, paved surfaces	GIS analysis	General health, mental health, common mental disorders	Semantic differential scale	(+)
Nishigaki, 2020 (*n* = 126,878) [26]	Vegetation cover types: trees vs. grass	GIS analysis	Depression	SGD	(+)
Wyles, 2019 (*n* = 4515) [86]	Environment types: coastal, rural green vs urban green	Self-reported	Restorativeness	Semantic differential scale	(+)
Reid, 2017 (*n* = 1387) [74]	Vegetation cover types: trees vs. grass	GIS analysis	Perceived health	Semantic differential scale	(+)
Jiang, 2020 (*n* = 212) [65]	Vegetation cover types: trees vs. low-lying vegetation	GIS analysis	General health; stress level	SF-12; PSS	(+)(–)
Egorov, 2020 (*n* = 186) [59]	Vegetation cover types: trees vs. grass	GIS analysis	Allostatic load	Clinically measured	(+)
Wheeler, 2015 (*n* = 31,672 LSOAs) [83]	Land cover diversity and environment types	SDI; GIS analysis	Health status	Semantic differential scale	(+)
Aerts, 2020 (*n* = 1872 census tracts) [34]	Land cover types: gardens, forests vs. grassland	GIS analysis	Respiratory diseases	Medication sales	(+)
Dennis, 2020 (*n* = 1673 LSOAs) [53]	Land cover diversity; vegetation cover types (ground, canopy vs. field-level)	SDI; GIS analysis	Chronic morbidity prevalence	CIDR	(+)
Sander, 2017 (*n* = 546 census blocks) [76]	Land cover types: water, forest, canopy, impervious surfaces, and grass	GIS analysis	BMI	Self-measured height & weight	(+)
Wu, 2017 (*n* = 543 districts) [33]	Vegetation cover types: forest, grassland, average tree canopy and near-road tree canopy	GIS analysis (50 m and 100 m buffers)	Autism	Medical records	(+)
Wu, 2018 (*n* = 187 census tracts) [85]	Land cover types: water, open land, developed land, barren land, forest, shrub land, grassland, agriculture and wetland	GIS analysis (50 m and 100 m buffers)	Sudden unexpected deaths	Medical records	(+)
Natural features (*n* = 15)					
Marselle, 2015 (*n* = 127) [22]	Perceived naturalness; bird, butterfly, plants and trees biodiversity	Semantic differential scale, manual counting of species	Positive and negative affect	PANAS scale	(–)
Astell-Burt, 2020 (*n* = 46,786) [47]	Tree coverage	GIS analysis	Diabetes, hypertension and cardiovascular diseases	Medical records	(+)
Wyles, 2019 (*n* = 4515) [86]	Protected/designated area status	Assigned by national agency	Restorativeness	Semantic differential scale	(+)
Leng, 2020 (*n* = 4155) [70]	Presence of evergreen trees	Environmental audits	Obesity, hypertension, diabetes, dyslipidaemia, stroke risk	Clinically measured BMI, blood pressure, blood glucose and lipid tests, stroke risk score card	(+)
Camargo, 2017 (*n* = 1392) [51]	Conditions of trees	Semantic differential scale	Quality of life	EUROHIS-QOL	(+)
Zhang, 2019 (*n* = 909) [24]	Tree density	POST	Quality of life	WHOQOL-BREF	(+)
Carter, 2014 (*n* = 440) [52]	Retention of green space and bushland	Semantic differential scale	Physical function	SF-36v2	(o)
Tan, 2019 (*n* = 326) [23]	Tree density	Environmental audits	Physical functioning, physical role, bodily pain and emotional role	SF-12v2	(o)
Pazhouhanfar, 2018 (*n* = 250) [73]	Tree and greening, flowers, sun, water, fresh air, and bird voice	Semantic differential scale	Mood ratings (relaxed/happy/excited/calmed)	Semantic differential scale	(+)
Zhu, 2020 (*n* = 240) [89]	Sky index, soft/hard surface ratio, vertical vegetation coverage	Grid pixel calculation	Restorative effect	PRS	(+)(–)
Wood, 2018 (*n* = 128) [44]	Ecological study richness score: plant diversity, bird diversity, bee/butterfly diversity, number of habitats	Environmental audits; SDI	Restorative effect	Modified ART	(+)
Honold, 2016 (*n* = 32) [62]	Diversity of vegetation: façade, design, building shapes, vanishing points, angles	Semantic differential scale	Stress level	Hair cortisol level (immunoassay)	(o)
Wheeler, 2015 (*n* = 31,672 LSOAs) [83]	Bird species richness, freshwater quality indicator, density of protected area density	Bird occurrence atlas, routine surface water testing	Health status	Semantic differential scale	(+)
Mears, 2020 (*n* = 345 LSOAs) [42]	Bird biodiversity	Citizen science programme data	Poor general health	Semantic differential scale	(o)
Lai, 2019 (*n* = 174 zip codes) [69]	Pollen allergenicity of trees	Street tree census	Asthma prevalence	Medical records	(–)
Infrastructure & amenities (*n* = 14)					
Droomers, 2015 (*n* = 48,132) [57]	Green intervention projects: reclaming vacant land, added recreational areas, paths and tracks, improved drainage, landscaping, maintenance	Construction and installation of new amenities	Health status	Semantic differential scale	(o)
Dobbinson, 2020 (*n* = 1670) [55]	Refurbishments to existing amenities: playground eqiupment, quality walking paths, shade and shade-sail	Construction and installation of new amenities	Positive and negative affect	PANAS scale	(o)
Wood, 2017 (*n* = 492) [84]	Park functions	POSDAT	Mental wellbeing	WEMWBS	(+)
McCarthy, 2017 (*n* = 13,469) [31]	Playground quality: useability, cleanliness and maintenance, distinct areas for different age groups, colourful eqiupment, shade cover, benches, fence, separation from roads	Environmental audits	BMI	Clinically measured	(o)
Rundle, 2013 (*n* = 13,102) [75]	Number of recreational facilities	Environmental audits	BMI	Clinically measured	(o)
Bojorquez, 2018 (*n* = 2345) [40] b	Park quality score: bathrooms, lighting, playground, etc. (9 items in total)	Environmental audits	Depressive symptoms	CES-D	(o)
Camargo, 2017 (*n* = 1392) [51]	Walking paths conditions	Semantic differential scale	Quality of life	EUROHIS-QOL	(+)
Zhang, 2019 (*n* = 909) [24]	Amenities: children’s play equipment, seating facilities, dog litter bags, water sources for dogs, drinking fountains, parking facilities, public transport, variety of permitted activities	POST	Quality of life	WHOQOL-BREF	(o)
Bai, 2013 (*n* = 893) [50]	Availability of facilities of interest	Semantic differential scale	BMI	Self-measured height and weight	(o)
Pope, 2018 (*n* = 578) [43]	Maintenance	Dichotomous survey question	Psychological distress	GHQ-12	(o)
Tan, 2019 (*n* = 326) [23]	Number of facilities and seats	Environmental audits	Physical functioning, physical role, bodily pain and emotional role	SF-12v2	(o)
Sugiyama, 2009 (*n* = 271) [25]	Quality of access paths	Semantic differential scale	Health statusQuality of life	No. of days with poor physical/mental healthSWLS	(o)
Mears, 2020 (*n* = 345 LSOAs) [32]	Play facilities: playgrounds, games area, skate or bike parks	Environmental audits	BMI	Clinically measured	(o)
Ngom, 2016 (*n* = N/A) [71]	Green space functions	GIS databases	Coronary heart disease, cerebrovascular disease, heart failure, diabetes, hypertension	Medical records	(+)
Size (*n* = 11)					
Wood, 2017 (*n* = 492) [84]	Park size	GIS analysis (1.6 km buffer)	Mental wellbeing	WEMWBS	(+)
Stark, 2014 (*n* = 44,282) [78]	Park size	GIS analysis (805 m buffer)	BMI	Self-measured height and weight	(+)
Rundle, 2013 (*n* = 13,102) [75]	Park size	GIS analysis (805 m buffer)	BMI	Clinically measured	(+)
Zhang, 2019 (*n* = 909) [24]	Park area	GIS analysis (400 m and 800 m buffers)	Quality of life	WHOQOL-BREF	(o)
Tan, 2019 (*n* = 326) [23]	Area	Environmental audits	Physical functioning, physical role, bodily pain and emotional role	SF-12v2	(+)
Kim, 2016 (*n* = 92) [30]	Size of tree canopy	GIS analysis (805 m buffer)	Quality of life	PedsQL	(+)
Kim, 2014 (*n* = 61) [29]	Size of tree canopy	GIS analysis (805 m buffer)	BMI	Clinically measured	(+)
Dennis, 2020 (*n* = 1673 LSOAs) [53]	Mean patch size	GIS databases	Chronic morbidity prevalence	CIDR	(+)
Wang, 2019 (*n* = 369 census tracts) [82]	Patch area	GIS analysis (805 m buffer)	All-cause, cardiovascular, chronic respiratory and neoplasm mortality	Medical records	(+)
Mears, 2020 (*n* = 345 LSOAs) [32]	Garden size	GIS analysis (300 m buffer)	Obesity rate	Clinically measured BMI	(o)
Mears, 2020 (*n* = 345 LSOAs) [42]	Garden size	GIS analysis (300 m buffer)	Poor general health Depression and severe mental illnesses	Semantic differential scale Medical records	(+)
Shape, pattern & connectivity (*n* = 8)					
Kim, 2016 (*n* = 92) [30]	Pattern of green space patches: fragmentation, shape irregularity, isolation from other patches	GIS analysis (805 m buffer)	Quality of life	PedsQL	(+)
Kim, 2014 (*n* = 61) [29]	Connectedness	GIS analysis (805 m buffer)	BMI	Clinically measured	(+)
Kim, 2021 (*n* = 2301 census tracts) [67]	Size & dispersion of tree canopy patches	GIS analysis	Asthma emergency visits	Medical records	(+)
Sander, 2017 (*n* = 546 census blocks) [76]	Contiguity	GIS analysis	BMI	Self-measured height and weight	(+)(–)
Wang, 2019 (*n* = 369 census tracts) [82]	Pattern of green space patches: fragmentation, connectedness, aggregation, shape irregularity	GIS analysis (805 m buffer)	All-cause, cardiovascular, chronic respiratory and neoplasm mortality	Medical records	(+)
Tsai, 2016 (*n* = 52 MSAs) [80]	Pattern of green space patches: aggregation, contrast between patch types	GIS analysis	BMI	Self-reported height and weight	(+)(–)
Jaafari, 2020 (*n* = 87 hexagons) [63]	Pattern of green space patches: patch density, connectedness, shape irregularity	GIS analysis	Mortality of respiratory cancer diseases and respiratory diseases	Medical records	(+)
Shen, 2017 (*n* = 48 districts) [77]	Pattern of green space patches: fragmentation, aggregation, between-patch distances	GIS analysis	Respiratory mortality	Medical records	(+)
Safety (*n* = 6)					
Orstad, 2020 (*n* = 3652) [72]	Perceived park crime	Dichotomous survey question	Mental health	Number of days with stress, depression, and emotion problems	(+)
Camargo, 2017 (*n* = 1392) [51]	Perceived safety of the way home	Semantic differential scale	Quality of life	EUROHIS-QOL 8-items	(+)
Bai, 2013 (*n* = 893) [50]	Safety	Semantic differential scale	BMI	Self-reported	(o)
Pope, 2018 (*n* = 578) [43]	Safety	Dichotomous survey question	Psychological distress	GHQ-12	(o)
Tan, 2019 (*n* = 326) [23]	Perceived safety: reduced visibility, prospect of crime, presence of security guards, fear of falling, unwell feelings	Survey questionnaire (details unspecified)	Physical functioning, physical role, bodily pain and emotional role	SF-12v2	(+)
Sugiyama, 2009 (*n* = 271) [25]	Safety: night-time safety, safety along surrounding paths, lack of crime	Semantic differential scale	Health status Quality of life	No. of days with poor physical/mental health SWLS	(+)
Cleanliness and absence of incivilities (*n* = 5)					
Stark, 2014 (*n* = 44,282) [78]	Cleanliness score	Parks Inspection Program audit tool	BMI	Self-measured height and weight	(+)
Rundle, 2013 (*n* = 13,102) [75]	Weeds, litter, glass, graffiti score and overall cleanliness score	Parks Inspection Program audit tool	BMI	Clinically measured	(o)
Zhang, 2019 (*n* = 909) [24]	Aesthetics: watered grass, no graffiti, no vandalism	POST	Quality of life	WHOQOL-BREF	(o)
Bai, 2013 (*n* = 893) [50]	Cleanliness	Semantic differential scale	BMI	Self-measured height and weight	(o)
Mears, 2020 (*n* = 345 LSOAs) [42]	Cleanliness	Environmental audits	Depression	Medical records	(+)
Peacefulness (*n* = 3)					
Herranz-Pascual, 2019 (*n* = 137) [61]	Soundscape characteristics	Semantic differential scale	Depression	Semantic differential scale	(+)
Sugiyama, 2009 (*n* = 271) [25]	NuisanceL dogs and dog foulings, presence of young people	Semantic differential scale	Health status Quality of life	No. of days with poor physical/mental health SWLS	(o)
Pazhouhanfar, 2018 (*n* = 250) [73]	Private environment	Semantic differential scale	Mood ratings (relaxed/happy/excited/calmed)	Semantic differential scale	(o)
Perceived quality/Satisfaction with quality (*n* = 7)					
Putra, 2020 (*n* = 4969) [36]	Perceived quality by parents	Semantic differential scale	Prosocial behaviour	SDQ	(+)
Feng, 2018 (*n* = 3897) [39]	Perceived quality	Dichotomous survey question	Psychological distress; serious mental illnesses	K6-PDS	(+)
Feng, 2019 (*n* = 3843) [38]	Perceived quality	Semantic differential scale	BMI	Self-measured height and weight	(+)
McEachan, 2018 (*n* = 805) [37]	Satisfaction with green space by parents	Semantic differential scale	Total difficulties, internalising difficulties, externalising difficulties and prosocial behaviours	SDQ	(+)
Bai, 2013 (*n* = 893) [50]	Attractiveness	Semantic differential scale	BMI	Self-measured height and weight	(o)
Pazhouhanfar, 2018 (*n* = 250) [73]	Attractiveness	Semantic differential scale	Mood ratings (relaxed/happy/excited/calmed)	Semantic differential scale	(o)
Jonker, 2014 (*n* = N/A)	Satisfaction with quality	Semantic differential scale	Life expectancy and healthy life expectancy	National life table data	(+)
Combination of features (*n* = 13)					
Zhang, 2017 (*n* = 223) [87]	Perceived quality: recreational facilities, amenities, natural features, absent of civilities, accessibility, maintenance	Semantic differential scale	Neighbourhood satisfaction	Semantic differential scale	(+)
Francis, 2012 (*n* = 911) [60]	Objective quality score: walking paths, shade, water features, irrigated lawn, birdlife, lighting, sporting facilities, playgrounds, type of surrounding roads, presence of nearby water Subjective quality score: atmosphere, comfort, safety, attractiveness and maintenance, variety of things to do, presence of adequate seating, public art, other people	POST (objective)Semantic differential scale (subjective)	Psychological distress	K6-PDS	(+)
Bird, 2016 (*n* = 380) [27]	Park typology: team sports features, pool-oriented features, perceived safety, cycling-oriented features, play area features, walking-oriented, aesthetically pleasing, incivilities, infrequent park installations, schoolyard features	Author-developed typology, with principal component analysis	% truncal fat	X-ray absorptiometry	(+)
Kruize, 2020 (*n* = 3947) [68]	Objective quality score: general characteristics, facilities, traffic safety, infrastructure, sidewalk amenities, incivilities Satisfaction with green space: quality, amount, maintenance, safety	Environmental audits/Semantic differential scale	Mental wellbeing	MHI-5	(+)
Vries, 2013 (*n* = 1641) [81]	Composite score: variation, maintenance, orderly arrangement, absence of litter, general impression	Semantic differential scale	Perceived general health; health complaints and mental health	SF-36; acute health-related complaint checklist; MHI-5	(+)
Dillen, 2012 (*n* = 1553) [54]	Green area quality: accessibility, maintenance, variation, naturalness, colourfulness, clear arrangement, shelter, absence of litter, safety, general impression	Environmental audits	Perceived general health; health complaints and mental health	SF-36; acute health-related complaint checklist; MHI-5	(+)
Carter, 2014 (*n* = 440) [52]	Useability: in good conditions, well-equipped, including spaces to relax and socialise	Semantic differential scale	General health and vitality	SF-36v2	(+)
Dzhambov, 2018 (*n* = 399) [58]	Perceived quality: safety, maintenance, aesthetic, suitability for sport and social interactions, biodiversity	Semantic differential scale	Mental health	GHQ-12	(+)
Tan, 2019 (*n* = 326) [23]	Aesthetics: colour, shape, diversity and seasonal variation of plants, maintenance, proportions of soft surfaces	Survey questionnaire (details unspecified)	Physical functioning	SF-12v2	(o)
Sugiyama, 2009 (*n* = 271) [25]	Pleasantness: adequacy for children to play, adequacy for adults to chat, variety of activities to engage in, quality of trees and plants, facilities (toilet, shelter)	Semantic differential scale	Health status Quality of life	No. of days with poor physical/mental health SWLS	(+)
Zhang, 2019 (*n* = 250) [88]	Visual sensation: Variety of plants, richness of plants’ colour, plant light and shadow mottle, nice road texture, rich terrain, wide view, ornamental water Auditory sensation: natural sound, sweet background music, happy people sounds (singing or playing instruments), quiet background, no traffic noise Tactile sensation: road material is comfortable and the foot feels good, strong hydrophilic, seat is comfortable for sitting, comfortable grass for flat lay	Semantic differential scale	Restorative effect	Semantic differential scale	(+)
Mears, 2020 (*n* = 345 LSOAs) [32]	Quality * Size ≥2 ha * Predominantly natural feeling * Good or better quality ratings from council assessment, based on: signage; provision of facilities; maintenance of paths; safety; planting and plant management; and cleanliness	Environmental audits	BMI	Clinically measured	(+)
Mears, 2020 (*n* = 345 LSOAs) [42]	Quality * Size ≥2 ha * Predominantly natural feeling * Good or better quality ratings from council assessment, based on: signage; provision of facilities; maintenance of paths; safety; planting and plant management; and cleanliness	Environmental audits	Poor general health	Semantic differential scale	(o)

Notes: Within each quality domain, studies were arranged by study design, and then by sample size. A full version of this table is available as Supplementary File S4. * Abbreviation: DA: dissemination areas; LSOA: lower layer Super output areas; MSA: metropolitan statistical areas ** AMI: acute myocardial infarction; ART: attention restoration theory; BMI: body mass index; CES-D: Center for Epidemiologic Studies-Depression; CIDR: comparative illness and disability ratio; CVD: cardiovascular diseases; EUROHIS-QOL-8: EUROHIS 8-item quality of life questionnaire; GDS: geriatric depression scale; GHQ-12: 12-item general health questionnaire; GIS: Geographic Information System; K10-PDS: Kessler ten-item psychological distress scale; K6-PDS: Kessler six-item psychological distress scale; MHI-5: 5-item mental health inventory; PANAS: positive and negative affect schedule; PedsQL: paediatric quality of life inventory; POST: Public Open Space Tool; POSDAT: Public Open Space Desktop Auditing Tool; PRS: perceived restorativeness scale; PSS: perceived stress scale; SDI: Shannon’s diversity index; SDQ: strengths and difficulties questionnaire; SF-8: eight-item short form survey; SF-12: 12-item short form survey; SF-12v2: short form 12 item (version 2); SF-36: 36-item short form survey; SF-36v2: short form 36 item (version 2); SWLS: satisfaction with life scale; WEMWBS: Warwick Edinburgh mental well-being scale; WHOQOL-BREF: World Health Organization quality-of-life scale. ≠ (+) Some evidence of protective associations; (–) some evidence of risk associations; (o) no significant associations observed.

## Data Availability

The data presented in this study are available in the main article and Supplementary Files.

## Supplementary Materials

The following are available online at https://www.mdpi.com/article/10.3390/ijerph182111028/s1, Supplementary File S1: Search strategy; Supplementary File S2: Quality assessment of included studies; Supplementary File S3: List of excluded studies from full-text review. Supplementary file S4: Summary of findings (expanded).
