# Supplementary material for: Green Space Quality and Health: A Systematic Review
Source: Int J Environ Res Public Health. 2021 Oct 20;18(21):11028. doi: 10.3390/ijerph182111028 (PMC8582763; doi:10.3390/ijerph182111028)
Supplement: Supplementary file 1 [file ijerph-18-11028-s001.zip › ijerph-1381739-supplementary/Green space quality and health - R1 - Supplementary file S1.pdf]

Green space quality and health: a systematic review (PY Nguyen et al, 2021)

Supplementary file S1. Search strategy

OVID – Last date searched: June 30th, 2021

1. MEDLINE(R) and Epub Ahead of Print, In-Process, In-Data-Review & Other Non-Indexed Citations, Daily and Versions(R) 1946 to June 30, 2021

2. Embase Classic + Embase to June 30, 2021

3. APA PsycInfo 1806 to June Week 4 2021

Health outcomes	**1**	Population Health/
	**2**	Maternal Health/ or Women's Health/
	**3**	Adolescent Health/
	**4**	Health/ or Health status/
	**5**	Urban Health/ or Environmental Health/
	**6**	(health adj1 (outcome* or stat*)).tw.
	**7**	Disease/ or chronic disease/ or Infections/
	**8**	(disease* or infecti* or disabilit* or disorder* or injur* or illness* or symptom*).tw.
	**9**	Pain/ or Pain Management/
	**10**	pain.tw.
	**11**	(death* or mortality or morbidit*).tw.
	**12**	Infant Death/ or Fetal Death/ or Perinatal Death/ or Maternal Death/ or maternal morbidity/ or newborn morbidity/ or perinatal morbidity/
	**13**	Death/ or Mortality/ or morbidity/
	**14**	Menopause/ or Perimenopause/ or Climacteric/
	**15**	(menopaus* or perimenopaus* or peri-menopaus* or premenopaus* pre-menopaus* or climacteric).tw.
	**16**	Menarche/ or Puberty/ or Pubarche/ or thelarche/ or spermatorrhea/
	**17**	Puberty, Precocious/
	**18**	((pubert* adj1 timing) or (sexual adj1 (precocity or prematurity)) or menarche or (precocious adj1 puberty) or (premature adj1 (pubarche or thelarche)) or (first adj1 spermatorrhea)).tw.
	**19**	Otitis Media/
	**20**	(otitis adj1 media).tw.
	**21**	Trematode Infections/ or Trematoda/ or Helminthiasis/
	**22**	(food-borne adj1 trematodias).tw.
	**23**	Pregnancy Complications/
	**24**	((maternal or pregnancy) adj2 (complication* or problem*)).tw.
	**25**	((pregnancy or birth) adj outcome*).tw.
	**26**	Pregnancy Outcome/
	**27**	Pre-Eclampsia/ or Obstetric Labor Complications/
	**28**	(obstructed and (labour or labor)).tw.
	**29**	Cesarean Section/ or Abortion, Therapeutic/ or Abortion, Spontaneous/ or Premature Birth/ or Infant, Premature/
	**30**	(abortion or miscarriage*).tw.
	**31**	(birth adj1 (asphyxia or trauma or sepsis)).tw.
	**32**	Birth Injuries/ or Neonatal Sepsis/ or Asphyxia Neonatorum/
	**33**	(birth adj1 weight).tw.
	**34**	Infant, Low Birth Weight/
	**35**	Infant Mortality/ or Maternal Mortality/

**36**	Infant, Premature, Diseases/ or Infant, Newborn, Diseases/
**37**	Developmental Disabilities/ or Congenital Abnormalities/ or Heart Defects, Congenital/
**38**	(congenital adj1 (anomal* or heart)).tw.
**39**	(neural adj1 tube adj1 defect*).tw.
**40**	Neural Tube Defects/
**41**	Infertility/ or Infertility, Female/ or Infertility, Male/ or impotence/
**42**	(fertil* or infertil* or impoten*).tw.
**43**	Malignant Neoplasm/ or Melanoma/ or Nonhodgkin Lymphoma/ or Leukemia/
**44**	(Cancer or Melanoma or lymphoma or leukemia or leukaemia or neoplasm*).tw.
**45**	Cardiovascular Disease/
**46**	(cardiac* or cardio* or vascula* or coronary).tw.
**47**	Cardiomyopathy/ or Myocarditis/ or Endocarditis/
**48**	(Cardiomyopath* or myocarditis or endocarditis).tw.
**49**	Atrial Fibrillation/ or Heart Atrium Flutter/ or heart rate variability/
**50**	((atrial adj1 (fibrillation or flutter*)) or (heart adj1 rate adj1 variability) or HRV).tw.
**51**	Aortic Aneurysm/ or Aorta Aneurysm/ or Cerebrovascular Accident/ or Transient Ischemic Attack/ or Heart muscle ischemia/ or Infarction/
**52**	((aort* adj1 aneurysm) or stroke or ischaem* or ischemi* or infarc*).tw.
**53**	Hypertension/
**54**	(hypertens* or (blood adj1 pressure)).tw.
**55**	Hyperlipidemia/ or Dyslipidemia/
**56**	(hyperlipid* or dyslipid*).tw.
**57**	Insulin Resistance/ or Diabetes Mellitus/
**58**	((insulin adj1 resistan*) or diabet* or (blood adj1 glucose) or insulin).tw.
**59**	Pneumoconiosis/ or Asthma/ or Chronic Obstructive Lung Disease/ or Emphysema/
**60**	(Pneumoconiosis or asthma* or COPD or emphysema).tw.
**61**	Respiratory Tract Disease/ or Respiratory Tract Infection/ or Lung Disease/
**62**	(respirat* or pulmonary).tw.
**63**	Liver Cirrhosis/ or Hepatitis/
**64**	(cirrho* or hepati*).tw.
**65**	Peptic Ulcer/ or Gastritis/ or Duodenitis/ or Appendicitis/ or Pancreatitis/ or digestive system disease/
**66**	((peptic adj1 ulcer*) or gastritis or duodenitis or appendicitis or pancreatitis or (digestive adj1 system)).tw.
**67**	Paralytic Ileus/ or Intestine Obstruction/ or Hernia/
**68**	((paralytic adj1 ileus) or (intestin* adj1 obstruct*) or hernia*).tw.
**69**	Alzheimer Disease/ or Dementia/
**70**	(Alzheimer* or dementia).tw.
**71**	Parkinson Disease/ or Multiple Sclerosis/ or Degenerative Disease/
**72**	(Parkinson* or degenerat* or (multiple adj1 sclerosis)).tw.
**73**	Migraine/ or Headache/
**74**	(migraine* or headache*).tw.
**75**	Schizophrenia/ or Epilepsy/
**76**	(Schizophren* or Epilep*).tw.
**77**	Glomerulonephritis/ or Urinary Tract Disease/ or Urinary Tract Infection/
**78**	(glomerulonephrit* or urinary).tw.
**79**	Hemoglobinopathy/ or Hemolytic Anemia/
**80**	(haemoglobinopath* or hemoglobinopath* or ((haemolytic or hemolytic) adj1 (anemia or anaemia))).tw.
**81**	Immunopathology/ or Autoimmune Disease/ or Inflammatory Disease/

	**82**	(autoimmun* or immun* or inflammat*).tw.
	**83**	Arthritis/ or Gout/
	**84**	(arthriti* or gout).tw.
	**85**	Drowning/
	**86**	drowning.tw.
	**87**	poisoning*.tw.
	**88**	(expos* adj3 (mechanical adj1 force*)).tw.
	**89**	(adverse effect* and treatment*).tw.
	**90**	(animal adj1 contact).tw.
	**91**	Life expectancy/ or quality adjusted life year/ or disability adjusted life year/
	**92**	((life adj1 expectan*) or (adjusted adj1 life adj1 year*) or daly* or qaly*).tw.
	**93**	Motor Skills Disorders/
	**94**	(motor adj1 development).tw.
	**95**	Hypochondriasis/
	**96**	Hypochondria*.tw.
	**97**	Thyroid disease/ or metabolic disorder/
	**98**	(Thyroid or metaboli*).tw.
	**99**	Osteoporosis/
	**100**	((bone adj1 density) or osteoporosis).tw.
	**101**	((physiological or physical or cognitive) adj1 function*).tw.
	**102**	Mental health/ or psychological well-being/ or emotional stress/ or anxiety/ or mood/ or mood disorder/ or mental disease/ or attention deficit disorder/ or depression/ or obsessive compulsive disorder/
	**103**	((mental adj1 (wellbeing or (well adj1 being))) or psycholog* or emotion* or mood*).tw.
	**104**	(Depress* or stress* or anxi* or fear* or frustrate* or lonel* or isolate* or panic or phobia).tw.
	**105**	(happiness or resilien* or optimis* or hope*).tw.
	**106**	(adhd or (attention adj1 deficit) or (obsessive adj1 compulsive) or OCD or aggress*).tw.
	**107**	(restorati* or empower*).tw.
	**108**	development.tw.
	**109**	body dysmorphic disorder/ or factitious disease/ or depersonalization/ or dissociative disorder/
	**110**	((body adj1 dysmorphic) or factitious or depersonali* or dissociative).tw.
	**111**	obesity/ or body mass/ or body weight/
	**112**	(obes* or (body adj1 mass) or BMI or (body adj1 weight) or adiposity or (waist adj1 circumference) or (waist adj2 hip) or (body adj1 fat) or skinfold or overweight or (over adj1 weight)).tw.
	**113**	nutritional deficiency/ or malnutrition/ or anorexia/
	**114**	((nutritional adj1 deficienc*) or malnutrition or malnourished or anorexi*).tw.
	**115**	or/1-114
**Green space quality**	**116**	Quality.tw.
	**117**	(Playground* or (play adj1 (ground* or space*))).tw.
	**118**	(Facilit* or amenit* or equipment or feature* or characteristic*).tw.
	**119**	(Clean* or rubbish* or trash* or litter* or graffiti).tw.
	**120**	(Maintain* or maintenance).tw.
	**121**	(Safe* or alcohol* or (drug adj1 use*)).tw.
	**122**	(Lighting or bright*).tw.
	**123**	(Nois* or quiet* or peace* or soundscape or acoustic*).tw.
	**124**	(Access* or walkab*).tw.
	**125**	((disabled or disability) adj1 friendl*).tw.
	**126**	(veget* or connect* or big or large or size or spacious or cheap or afford* or usab* or bicycle* or cycl* or parking or dirt*).tw.

	**127**	((vegetation or tree* or plant* or shrub* or grass* or turf or bush* or canopy or understory or ((ground or land) adj1 cover) or undercover or habitat or streetscape) adj25 (coverage or type* or species or divers* or biodiver* or variety or variation* or richness or compar* or color* or colour* or allergenic* or dense or density or visibility or (view adj3 obstruct*))).tw.
	**128**	(tree* adj25 (shrub* or grass* or understory or (ground adj1 cover) or undercover or bush* or turf)).tw.
	**129**	(background adj5 (sound* or music or traffic or nois*)).tw.
	**130**	((path* or road* or sidewalk*) adj5 (material* or quality or walkab* or texture or surface* or impervious)).tw.
	**131**	(wilderness or naturalness).tw.
	**132**	(shade* or shelter*).tw.
	**133**	(ornament* or fountain* or ((water or freshwater) adj3 (cover or source* or taps or open))).tw.
	**134**	(bird* or butterf* or bees or wildlife or animal*).tw.
	**135**	((art* or historic* or social or cultur*) adj5 (public or galler* or display* or sculptur* or statue* or install*)).tw.
	**136**	(seats or seat or sitting).tw.
	**137**	toilet*.tw.
	**138**	(naturalness or (natural adj2 (feel* or sensation))).tw.
	**139**	((walk* or cycl* or sport* or skat* or biking or bike*) adj5 ((feature* and amenit*) or facilit* or equipment or install*)).tw.
	**140**	((private or privacy) adj5 (environment* or atmosphere* or setting*)).tw.
	**141**	(connectedness or connectivity or contigu* or fragment* or aggregate* or (shape adj3 (regular* or irregular*))).tw.
	**142**	((protected or designated) adj1 area*).tw.
	**143**	vandalis*.tw.
	**144**	or/116-143
**Green space types**	**145**	(Wilderness or ((wild or natural or municipal or community or public or open) adj1 (land or park or parks or garden*))).tw.
	**146**	(greenspace or greenness or greener* or (green adj1 (space* or infrastructure))).ti,ab,tw.
	**147**	((municipal or botanic or city or urban) adj1 park*).tw.
	**148**	(urban adj3 (park* or garden* or parkland*or horticultur* or botanic* or arboretum or Allotment)).tw.
	**149**	((natural or green or wild) adj2 (facilit* or area*)).tw.
	**150**	((trail* or belt*) adj3 (recreation or green or cycl* or walk*)).tw.
	**151**	national park/ or recreational park/
	**152**	(greenspace or ((green or open or public) adj1 space*) or park or parks).tw.
	**153**	or/145-152
	**154**	115 and 144 and 153
	**155**	NOT (exp animals/ not humans.sh.)

4. Scopus – Date last search: June 30th, 2021

Health outcomes	TITLE-AB S-KEY (("Maternal Health" OR "Women's Health" OR "Adolescent Health" OR "Health status" OR "Urban Health" OR "Environmental Health" OR "health outcome*" OR "health stat*" OR "disease*" OR abnormalit* OR infecti* OR disabilit* OR disorder* OR injur* OR illness* OR symptom* OR pain OR death* OR mortality OR morbidit* OR "Infant Death*" OR "Fetal Death*" OR "Perinatal Death*" OR "Maternal Death*" OR morbidit* OR mortality OR menopaus* OR perimenopaus* OR peri-menopaus* OR premenopaus* AND pre-menopaus* OR climacteric OR "pubert* timing" OR "sexual precocity" OR "sexual prematurity" OR menarche OR "precocious puberty" OR pubarche OR thelarche OR "first spermatorrh*" OR "otitis media" OR "trematod*" OR "helminth*" OR "maternal complication*" OR "pregnancy complica*" OR "maternal problem*" OR "birth complicat*" OR ((pregnancy OR birth) W/1 outcome*) OR pre-eclampsia OR preeclampsia OR eclapmsia OR "obstructed W/1 (labour OR labor)" OR "C-section" OR "caesarean" OR "abort*" OR "miscarriage*" OR premature OR "birth asphyxia" OR "birth trauma" OR "birth sepsis" OR "neonatal sepsis" OR "asphyxia neonatorum" OR "birth weight" OR "heart defect*" OR "congenital anomal*" OR "congenital heart" OR "neural tube defect*" OR fertil* OR infertil* OR impoten* OR "cancer*" OR "neoplasm*" OR melanoma OR "non-Hodgkin lymphoma" OR "leuk*mia" OR cardiac* OR cardio* OR vascula* OR coronary OR myocarditis OR endocarditis OR "Atrial Fibrillation" OR "atrial flutter*" OR "heart rate variability" OR hrv OR "Aort* Aneurysm" OR cerebrovascula* OR stroke OR ischaem* OR ischemi* OR infarc* OR hypertens* OR "blood pressure" OR hyperlipid* OR dyslipid* OR "insulin resistan*" OR "diabet*" OR "blood glucose" OR insulin OR "Pneumoconiosis" OR asthma* OR copd OR emphysema OR respirat* OR pulmonary OR cirrho* OR hepati* OR "Peptic Ulcer*" OR gastritis OR duodenitis OR appendicitis OR pancreatitis OR "digestive system" OR "paralytic ileus" OR "intestin* obstruct*" OR hernia* OR alzheimer* OR dementia OR parkinson* OR degenerat* OR "multiple sclerosis" OR migraine* OR headache* OR schizophren* OR epilep* OR glomerulonephrit* OR urinary OR h*moglobinopath* OR h*molytic OR anaemi* OR anemi* OR autoimmun* OR immun* OR inflammat* OR arthriti* OR gout OR drown* OR poisoning* OR (expos* W/3 "mechanical force*") OR "adverse effect*" OR treatment* OR "animal contact" OR "life expectan*" OR "adjusted life year*" OR daly* OR qaly* OR "Motor development" OR hypochondria* OR thyroid OR metaboli* OR "bone density" OR osteoporo* OR physiological OR "physical function*" OR "cognitive function*" OR psycholog* OR meantal OR emotion* OR mood* OR depress* OR stress* OR anxi* OR fear* OR frustrate* OR lonel* OR isolate* OR panic OR phobia OR happiness OR resilien* OR optimis* OR hope* OR adhd OR "attention deficit" OR "obsessive-compulsive" OR ocd OR aggress* OR restorati* OR empower* OR development OR "body dysmorphic" OR "factitious" OR depersonali* OR "dissociative" OR obes* OR "body mass" OR bmi OR "body weight" OR adiposity OR "waist circumference" OR "waist to hip" OR "body fat" OR skinfold OR overweight OR "over-weight" OR nutriti* OR malnutrition OR malnourished OR anorexi*)
Green space quality	AND (quality AND (characteristic* OR feature* OR playground* OR "play-ground*" OR "play ground*" OR "play space*" OR facilit* OR amenit* OR equipment OR clean* OR rubbish* OR trash* OR litter* OR dirt* OR graffiti OR maintain* OR maintenance OR safe* OR alcohol* OR "drug use*" OR lighting OR bright* OR nois* OR quiet* OR peace* OR soundscape OR acoustic* OR access* OR walkab* OR "disabled-friendl*" OR "disability-friendl*" OR "disabled friendl*" OR "disability friendl*" OR veget* OR connect* OR big OR large OR size OR spacious OR cheap OR afford* OR usab* OR bicycle* OR cycl* OR parking OR ((vegetation OR tree* OR plant* OR shrub* OR grass* OR turf OR bush* OR canopy OR understory OR ((ground OR land) W/1 cover) OR undercover OR habitat OR streetscape) W/25 (coverage OR type* OR species OR divers* OR biodiver* OR variety OR variation* OR richness OR compar* OR color* OR colour* OR allergenic* OR dense OR density OR visibility OR (view W/3 obstruct*))) OR (tree* W/25 (shrub* OR grass* OR understory OR (ground W/1 cover) OR undercover OR bush* OR turf)) OR (background W/5 (sound* OR music OR traffic OR nois*)) OR ((path* OR road* OR sidewalk*) W/5 (material* OR quality OR walkab* OR texture OR surface* OR impervious)) OR wilderness OR naturalness OR shade* OR shelter* OR ornament* OR fountain* OR ((water OR freshwater) W/3 (cover OR source* OR taps OR open)) OR bird* OR butterf* OR bees OR wildlife OR animal* OR ((art* OR historic* OR social OR cultur*) W/5 (public OR galler* OR display* OR sculptur* OR statue* OR install*)) OR seats OR seat OR sitting OR toilet* OR (natural W/2 (feel* OR sensation)) OR ((walk* OR cycl* OR sport* OR skat* OR biking OR bike*) W/5 ((feature* AND amenit*) OR facilit* OR equipment OR install*)) OR ((private OR privacy) W/5 (environment* OR atmosphere* OR setting*)) OR (connectedness OR connectivity OR contigu* OR fragment* OR aggregate* OR (shape W/3 (regular* OR irregular*))) OR ((protected OR designated) W/1 area*) OR vandalis*))
Green space types	AND (greenspace OR "green-space" OR "green space" OR "wild land" OR botanic* OR "natural land" OR "natural space" OR "wild space" OR garden* OR horticult* OR "shinrin-yoku" OR tree* OR jungle* OR forest* OR woodland* OR (green AND (trail* OR belt* OR corridor*))) OR park OR parks OR parkland)

5. CINALH Plus via EBSCO – Last date searched: June 30th, 2021

Health outcomes	S1	(MH "Population Health+") OR (MH “Maternal-Child Health+") OR (MH “Adolescent Health+") OR (MH "Health Status+") OR (MH "Urban Health+") OR(MH "Environmental Health+") OR (MH “Outcomes (Health Care)+") OR (AB "health outcome*") OR (AB "healthstat*") OR (MH "Chronic Disease+") OR (MH "Acute Disease+") OR (AB "pain")
	S2	(AB "death*") OR (AB "mortality") OR (AB "morbidit*") OR (MH “Mortality+") OR (MH "Infant Death+") OR (MH "Perinatal Death+") OR (MH “Menopause, Premature+") OR (MH “Andropause+") OR (MH “Postmenopausal Disorders+") OR (MH “Premenopause+") OR (MH “Perimenopause+") OR (MH “Menopause+")
	S3	(MH "Climacteric+") OR (AB "menopaus*") OR (AB "perimenopaus*") OR (AB "peri-menopaus*") OR (AB "premenopaus*") OR (AB "premenopaus*") OR (AB "climacteric") OR (MH "Menarche+") OR (AB "Menarche") OR (MH "Puberty, Precocious+") OR (MH "Puberty, Delayed+") OR (AB "pubert* timing")
	S4	(AB "sexual precocity") OR (AB "sexual prematurity") OR (AB "precocious puberty") OR (AB "premature puberty") OR (AB "premature pubarche") OR (AB "premature thelarche") OR (AB "firstspermatorr*") OR (MH "Otitis Media+") OR (AB "Otitis Media") OR (MH “Trematode Infections+") OR (AB "Trematod*") OR (MH "Helminthiasis+")
	S5	(AB "Helminthiasis") OR (MH “Pregnancy Complications+") OR (MH “Labor Complications+") OR ((AB (maternal or pregnancy or gestation*) and (issue* or complication* or problem* or outcome* or adverse)) OR (MH "Pre-Eclampsia+") OR (MH "Eclampsia+") OR (MH "Pregnancy-Induced Hypertension+") OR (AB "Pre-Eclampsia") OR (AB "Eclampsia") OR (AB "obstruct* labor") OR (AB "obstruct* labour") OR (MH "Cesarean Section+")
	S6	(AB "Cesarean Section") OR (AB "C-Section") OR (MH "Abortion, Spontaneous+") OR (MH "Abortion, Induced+") OR (AB "abortion")
	S7	(MH "Neonatal Sepsis+") OR ((MH “Congenital, Hereditary, and Neonatal Diseases and Abnormalities+")) OR (MH "Asphyxia Neonatorum+") OR (MH “Infant, Premature, Diseases+") OR (MH "Infant, Newborn, Diseases+") OR (MH "Infant, Premature+") OR (AB "birthasphyxia") OR (AB "birth trauma") OR (AB "birth sepsis") OR (AB "low birthweight") OR (AB "birth injur*") OR (MH “Developmental Disabilities+")
	S8	(AB "congenital anomal*") OR (AB "congenital heart") OR (AB "neural tube defect*") OR (AB "fertil*") OR (AB "infertil* ") OR (AB "impoten*") OR (MH “Infertility+") OR (MH "Neoplasms+") OR (AB "Cancer") OR (AB "Melanoma") OR (AB "non-Hodgkin lymphoma") OR (AB "leuk*mia")
	S9	(AB "neoplasm") OR (MH “Cardiovascular Diseases+") OR ((AB ((cardiac or cardio* or vascula* or coronary) and (disease* or disorder* or condition* or illness* or syndrome* or dysfunc* or disabilit* or injur* or symptom*))) OR (AB "cardiomyopath*") OR (AB "myocarditis") OR (AB "endocarditis") OR (AB "atrialfibrillation") OR (AB "atrial flutter") OR (AB "heart rate variability") OR (MH “Motor Neuron Diseases+") OR (MH “Nervous System Diseases+") OR (MH “Digestive System Diseases+")
	S10	(MH "Musculoskeletal Diseases+") OR (MH "Respiratory Tract Diseases+") OR ((MH "Nutritional and Metabolic Diseases+")) OR (MH "Endocrine Diseases+") OR (MH "Immunologic Diseases+") OR (MH "Male Urogenital Diseases+") OR ((MH "Female Urogenital Diseases and Pregnancy Complications+")) OR ((MH "Skin and Connective Tissue Diseases+"))
	S11	(AB ((neuro* or "nervous system") and (disease* or disorder* or condition* or illness* or syndrome* or dysfunc* or disabilit* or injur* or symptom*))
	S12	(AB ((urinary or urogenital) and (disease* or disorder* or condition* or illness* or syndrome* or dysfunc* or disabilit* or injur* or symptom*))
	S13	(AB (respirat* and (disease* or disorder* or condition* or illness* or syndrome* or dysfunc*))
	S14	(AB (immun* and (disease* or disorder* or condition* or illness* or syndrome* or dysfunc*))
	S15	(AB (digestive and (disease* or disorder* or condition* or illness* or syndrome* or dysfunc* or disabilit* or injur* or symptom*))
	S16	(AB ((muscular* or musculoskelet*) and (disease* or disorder* or condition* or illness* or syndrome* or dysfunc*))
	S17	(AB (metaboli* and (disease* or disorder* or condition* or illness* or syndrome* or dysfunc* or disabilit* or injur* or symptom*))
	S18	(AB (endocrine and (disease* or disorder* or condition* or illness* or syndrome* or dysfunc*))
	S19	(AB (thyroid* and (disease* or disorder* or condition* or illness* or syndrome* or dysfunc*))
	S20	(AB "infecti*") OR (AB "stroke") OR (AB "ischaem*") OR (AB "ischemi*") OR (AB "infarc*") OR (AB "hypertens*") OR (AB "blood pressure") OR (AB "hyperlipid*") OR (AB "dyslipid*") OR (AB "insulin resistan*") OR (AB "diabet*") OR (AB "blood glucose") OR (AB "insulin") OR (AB "Pneumoconiosis") OR (AB "asthma*") OR (AB "COPD")
	S21	(AB (hepati* and (disease* or disorder* orcondition* or illness* or syndrome* ordysfunc* or disabilit* or injur* or symptom*))
	S22	(AB "emphysema") OR (AB ("cirrho*" or hepatitis)) OR (AB "peptic ulcers") OR (AB "Gastritis") OR (AB "duodenitis") OR (AB "appendicitis") OR (AB "pancreatitis") OR (AB "paralyticileus") OR ((AB ("intestin*" and"obstruct*")) OR (AB "hernia") OR (AB "Alzheimer*")

	**S23**	(AB "dementia") OR (AB "Parkinson*") OR (AB "degenerat*") OR (AB "multiple sclerosis") OR (AB "migraine*") OR (AB "headache*") OR (AB "Schizophren*") OR (AB "Epilep*") OR (AB "glomerulonephrit*") OR (AB "h*moglobinopath*") OR (AB "anaemi*") OR (AB "anemi*")
	**S24**	(AB "autoimmun*") OR (AB "inflammat*") OR (AB "arthriti*") OR (AB "gout") OR (AB "drowning") OR (AB "poison*") OR (AB "adverse effect*") OR (AB "animal contact") OR (MH “Quality-Adjusted Life Years+") OR (MH “Disability-Adjusted Life Years+") OR (MH "Quality of Life+") OR (MH "Life Expectancy+")
	**S25**	(AB "autoimmun*") OR (AB "inflammat*") OR (AB "arthriti*") OR (AB "gout") OR (AB "drowning") OR (AB "poison*") OR (AB "adverse effect*") OR (AB "animal contact") OR (MH “Quality-Adjusted Life Years+") OR (MH “Disability-Adjusted Life Years+") OR (MH "Quality of Life+") OR (MH "Life Expectancy+")
	**S26**	(MH "Psychosocial Aspects of Illness+") OR ((MH "Behavioral and Mental Disorders+")) OR ((AB (depress* or stress* or anxi* or fear* or frustrate* or lonel* or panic or phobia)) OR ((AB (happiness or resilien* oroptimis* or hope*)) OR ((AB (adhd or"attention deficit" or "obsessivecompulsive" or OCD or aggress*)) OR ((AB (restorati* or empower*)) OR (AB "body dysmorphic") OR (AB "factitious") OR (AB "depersonali* ") OR (AB "dissociative") OR (MH "Obesity+") OR (MH "Thinness+")
	**S27**	((AB "mental wellbeing" or "mentalwell-being" or psycholog* or emotion* ormood*")) OR (AB "obes*") OR (AB "body mass") OR (AB "BMI") OR (AB "body weight") OR (AB "adiposity") OR (AB "overweight") OR (AB "overweight") OR (AB "under-weight") OR (AB "nutritional deficiency") OR (AB "malnutrition") OR (AB "anorexi*") OR (AB "malnourished")
	**S28**	(S1 OR S2 OR S3 OR S4 OR S5 OR S6 OR S7 OR S8 OR S9 OR S10 OR S11 OR S12 OR S13 OR S14 OR S15 OR S16 OR S17 OR S18 OR S19 OR S20 OR S21 OR S22 OR S23 OR S24 OR S25 OR S26 OR S27)))
**Green space quality**	**S29**	(AB "Quality")
	**S30**	(AB "Playground* ") OR (AB "playground*") OR (AB "play ground*") OR (AB "play ground*") OR (AB "Facilit*") OR (AB "amenit*") OR (AB "equipment") OR (AB "Clean*") OR (AB "rubbish*") OR (AB "trash*") OR (AB "litter*") OR (AB "graffiti")
	**S31**	(AB "Maintain*") OR (AB "maintenance") OR (AB "Safe*") OR (AB "alcohol*") OR (AB "drug use*") OR (AB "Lighting") OR (AB "bright*") OR (AB "drug addict*") OR (AB "Nois*")
	**S32**	(AB "acoustic*") OR (AB "Access*") OR (AB "walkab*") OR (AB "disabledfriendl*") OR (AB "disability-friendl*") OR (AB "disabled friendl*") OR (AB "disability friendl*") OR (AB "veget*") OR (AB "connect*") OR (AB "large") OR (AB "size*") OR (AB "spacious")
	**S33**	(AB "cheap") OR (AB "afford*") OR (AB "usab*") OR (AB "bicycle*") OR (AB "cycl*") OR (AB "parking") OR (AB "dirt*")
	**S34**	AB (vegetation or tree* or plant* or shrub* or grass* or turf or bush* or canopy or understory or ((ground or land) N1 cover) or undercover or habitat or streetscape) N25 (coverage or type* or species or divers* or biodiver* or variety or variation* or richness or compar* or color* or colour* or allergenic* or dense or density or visibility or (view N3 obstruct*)))
	**S35**	AB (tree* N25 (shrub* or grass* or understory or (ground N1 cover) or undercover or bush* or turf))
	**S36**	AB (background N5 (sound* or music or traffic or nois*))
	**S37**	AB ((path* or road* or sidewalk*) N5 (material* or quality or walkab* or texture or surface* or impervious))
	**S38**	(AB “wilderness”) or (AB “ (AB “naturalness”) or (AB “ (AB “shade*”) or (AB “ (AB “shelter*”) or (AB “nament*) or (AB “ fountain*)
	**S39**	AB ((water or freshwater) N3 (cover or source* or taps or open))
	**S40**	AB ((bird* OR butterf* OR bees OR wildlife OR animal*) AND (diversity or biodiversity))
	**S41**	AB (art* or historic* or social or cultur*) N5 (public or galler* or display* or sculptur* or statue* or install*
	**S42**	AB natural N2 (feel* or sensation)
	**S43**	AB ((walk* or cycl* or sport* or skat* or biking or bike*) N5 ((feature* or amenit* or facilit* or equipment or install*))
	**S44**	AB ((private or privacy) N5 (environment* or atmosphere* or setting*))
	**S45**	AB (connectedness or connectivity or contigu* or fragment* or aggregate* or (shape N3 (regular* or irregular*)))
	**S46**	(AB ((protected or designated) N1 area*)
	**S47**	AB vandalis*
	**S48**	S29 or s30 or s31 or s32 or s33 or s34 or s35 or s36 or s37 or s38 or s39 or s40 or s41 or s42 or s43 or s44 or s45 or s46 or s47

Green space types	S49	(AB "greenspace") OR (AB "greenspace") OR (AB "greenness") OR (AB "greener*") OR (AB "green infrastructure") OR (AB "Wilderness") OR (AB "wild land") OR (AB "natural land") OR (AB "municipal land") OR (AB "community land") OR (AB "public land") OR (AB "open land")
	**S50**	(AB "municipal space") OR (AB "natural space") OR (AB "wild space") OR (AB "open space") OR (AB "parks") OR (AB "park") OR (AB "parkland") OR ((AB (urban and (botanic* or arboretum or horticultur* or allotment)) OR (AB "public garden*") OR ((AB (natural and facilit*)) OR (AB "natural area*") OR (AB "green area*")
	**S51**	(AB "wild area*") OR ((AB (trail* or belt*) and (recreation* or green* or cycl* or walk*)) OR (AB "woodland*") OR (AB "shinrin-yoku")
	**S52**	S49 OR S50 OR S51
	**S53**	S28 AND S48 AND S52
