# Supplementary material for: Green Space Quality and Health: A Systematic Review
Source: Int J Environ Res Public Health. 2021 Oct 20;18(21):11028. doi: 10.3390/ijerph182111028 (PMC8582763; doi:10.3390/ijerph182111028)
Supplement: Supplementary file 1 [file ijerph-18-11028-s001.zip › ijerph-1381739-supplementary/Green space quality and health - R1 - Supplementary file S2.pdf]

Green space quality and health: a systematic review (PY Nguyen et al, 2021)

Supplementary file S2. Quality assessment of included studies

For the list of questions assessed for each type of studies, see https://www.nhlbi.nih.gov/health-topics/study-quality-assessment-tools

For ecological studies, the adapted questions are:

Q14 (modified): Were key potential confounding variables measured at the ecological unit level and adjusted statistically for their impact on the relationship between exposure(s) and outcome(s)?

Q15 (added): Was spatial autocorrelation addressed?

Q16 (added): Was variation of outcome distribution within each unit of analysis accounted for in any way?

Controlled intervention studies (quasi-experimental)

Study	Q1	Q2	Q3	Q4	Q5	Q6	Q7	Q8	Q9	Q10	Q11	Q12	Q13	Q14	Total score	Total applicable	Adjusted score
Dobbinson_2020	N	N/A	Y	N	N	Y	N/R	N/R	Y	Y	Y	N	Y	Y	7	13	0.54
Droomers_2015	N	N/A	Y	N	N	N/R	N	N/R	Y	Y	N	N	Y	Y	5	13	0.38
Zhang_2017	N	N/A	Y	N	N	N	N	N	Y	Y	N	N	Y	Y	5	13	0.38

Before-after studies

Study	Q1	Q2	Q3	Q4	Q5	Q6	Q7	Q8	Q9	Q10	Q11	Q12	Total score	Total applicable	Adjusted score
Marselle_2015	Y	Y	N	N	N	Y	Y	N	N/A	N	N	N/A	4	10	0.40

Case-controlled studies (case-crossover)

Study	Q1	Q2	Q3	Q4	Q5	Q6	Q7	Q8	Q9	Q10	Q11	Q12	Total score	Total applicable	Adjusted score
Stas_2021	Y	Y	N	Y	Y	Y	N	Y	N	Y	Y	Y	9	12	0.75

Observational cohort and cross-sectional studies

Study	Q1	Q2	Q3	Q4	Q5	Q6	Q7	Q8	Q9	Q10	Q11	Q12	Q13	Q14	Total score	Total applicable	Adjusted score
Astell-Burt_2019	Y	Y	N	Y	Y	N	N	N/A	Y	N	Y	Y	N	Y	7	13	0.54
Astell-Burt_2020a	Y	Y	N	Y	Y	N	N	N/A	Y	N	Y	Y	N	Y	7	13	0.54
Astell-Burt_2020b	Y	Y	N	Y	Y	N	N	N/A	Y	N	Y	Y	N	Y	7	13	0.54

Study	Q1	Q2	Q3	Q4	Q5	Q6	Q7	Q8	Q9	Q10	Q11	Q12	Q13	Q14	Total score	Total applicable	Adjusted score
Astell-Burt_2020c	Y	Y	N	Y	Y	N	N	N/A	Y	N	N	Y	N	Y	6	13	0.46
Astell-Burt_2021	Y	Y	N	Y	Y	N	N	N/A	Y	N	Y	Y	N	Y	7	13	0.54
Bai_2013	Y	Y	N	N	N	N	N	N/A	Y	N	N	N	N/A	Y	3	12	0.25
Bird_2016	Y	Y	N	Y	N	N	N	N/A	Y	Y	Y	Y	Y	Y	8	13	0.62
Bojorquez_2018	Y	Y	Y	Y	N	N	N	Y	Y	N	Y	N	N/A	Y	7	13	0.54
Camargo_2017	Y	Y	Y	Y	N	N	N	N/A	N	N	Y	N	N/A	Y	5	12	0.42
Carter_2014	Y	Y	N	Y	N	N	N	N/A	Y	N	Y	N	N/A	Y	5	12	0.42
Dillen_2012	Y	Y	N	Y	N	Y	N	N/A	N	N	Y	Y	N/A	Y	6	12	0.50
Donovan_2018	Y	Y	Y	Y	N	N	N	N/A	Y	N	Y	Y	N/R	Y	7	13	0.54
Dzhambov_2018	Y	Y	Y	Y	N	N	N	N/A	N	N	Y	N	N/A	Y	5	12	0.42
Egorov_2020	Y	Y	N/A	Y	N	Y	Y	Y	Y	N	Y	Y	N/A	Y	9	12	0.75
Feng_2018	Y	Y	Y	Y	N	N	N	N/A	N	Y	Y	N	Y	Y	7	13	0.54
Feng_2019	Y	Y	Y	Y	N	N	N	N/A	N	N	N	N	Y	Y	5	13	0.38
Francis_2012	Y	Y	N	Y	N	Y	Y	Y	Y	N	Y	N	Y	Y	9	14	0.64
Gernes_2019	Y	Y	Y	Y	N	N	N	N/A	Y	N	Y	Y	Y	Y	8	13	0.62
Herranz-Pascual_2019	Y	Y	N/A	Y	N	N	N	N/A	N	N	N	Y	N/A	Y	4	11	0.36
Honold_2016	Y	Y	N	Y	Y	Y	Y	N/A	Y	N	Y	Y	N/A	Y	9	12	0.75
Jarvis_2020	Y	Y	Y	Y	N	N	N	N/A	Y	N	N	Y	N/A	Y	6	12	0.50
Jiang_2020	Y	N	N/R	Y	N	N	N	N/A	Y	N	Y	Y	N/A	Y	5	12	0.42
Kim_2014	Y	Y	N/R	Y	N	N	N	N	Y	N	Y	Y	N/A	Y	6	13	0.46
Kim_2016	Y	Y	N/R	Y	N	N	N	N	Y	N	N	Y	N/A	Y	5	13	0.38
Kruize_2020	Y	Y	N	Y	N	N	N	N/A	Y	N	Y	Y	N/A	Y	6	12	0.50
Leng_2020	Y	N	N/R	Y	N	N	N	N/A	Y	N	Y	Y	N/A	Y	5	12	0.42
McCarthy_2017	Y	Y	Y	Y	N	N	N	Y	Y	N	Y	N	N/A	Y	7	13	0.54
McEachan_2018	Y	Y	Y	Y	N	N	N	N/A	N	N	Y	N	Y	Y	6	13	0.46
Nishigaki_2020	Y	Y	Y	Y	N	Y	Y	N/A	Y	N	Y	Y	N/A	Y	9	12	0.75
Orstad_2020	Y	Y	Y	Y	N	N	N	N/A	Y	N	N	N	N/A	Y	5	12	0.42
Parmes_2020	Y	Y	Y	N	N	N	N	N/A	Y	N	N	Y	N	Y	5	13	0.38
Pazhouhanfar_2018	Y	N	N/A	Y	N	N	N	N/A	N	N	Y	N	N/A	N	2	11	0.18
Pope_2018	Y	Y	N	Y	Y	N	N	N/A	N	N	Y	Y	N/A	Y	6	12	0.50
Putra_2020	Y	Y	Y	Y	N	N	N	N/A	N	N	Y	N	N/R	Y	5	13	0.38
Reid_2017	Y	N	N	Y	N	N	N	N	Y	N	N	Y	N/A	Y	4	13	0.31
Richardson_2018	Y	Y	N/A	Y	N	N	N	N/A	N	N	Y	Y	Y	Y	6	12	0.50
Rundle_2013	Y	Y	N/A	Y	N	N	N	Y	Y	Y	Y	Y	N/A	Y	8	12	0.67
Stark_2014	Y	Y	Y	Y	N	N	N	Y	Y	Y	N	Y	N/A	Y	8	13	0.62
Sugiyama_2009	Y	N	N	Y	N	N	N	Y	Y	N	N	N	N/A	Y	4	13	0.31

Study	Q1	Q2	Q3	Q4	Q5	Q6	Q7	Q8	Q9	Q10	Q11	Q12	Q13	Q14	Total score	Total applicable	Adjusted score
Tan_2019	Y	Y	N	Y	N	N	N	N	Y	N	Y	Y	N/A	Y	6	13	0.46
Vries_2013	Y	Y	N	Y	N	Y	N	N/A	Y	N	Y	Y	N/A	Y	7	12	0.58
Wood_2017	Y	Y	N	Y	N	Y	Y	Y	Y	N	Y	Y	N	Y	9	14	0.64
Wood_2018	Y	Y	N/A	Y	N	N	N	N/A	Y	Y	Y	Y	N/A	Y	7	11	0.64
Wyles_2019	Y	Y	N	Y	N	Y	Y	N/A	Y	N	N	N	N/A	Y	6	12	0.50
Zhang_2019a	Y	Y	N/R	Y	N	N	N	N/A	Y	N	Y	N	N/A	Y	5	12	0.42
Zhang_2019b	Y	Y	Y	Y	N	N	N	N/A	Y	N	Y	Y	N/A	Y	7	12	0.58
Zhu_2020	Y	Y	Y	Y	N	N	N	N	Y	N	Y	N	N/A	N	5	13	0.38

Ecological studies

Study	Q1	Q2	Q3	Q4	Q5	Q6	Q7	Q8	Q9	Q10	Q11	Q12	Q13	Q14	Q15	Q16	Total score	Total applicable	Adjusted score
Aerts_2020	Y	Y	N/A	Y	N	N	N	N	Y	N	Y	Y	N/A	Y	Y	N	7	14	0.50
Dennis_2020	Y	Y	N/A	Y	N	N	N	N	Y	N	Y	Y	N/A	Y	Y	Y	8	14	0.57
Jaafari_2020	Y	N	N/A	Y	N	N	N	N/A	N	N	Y	Y	N/A	N	N	N	3	13	0.23
Jonker_2014	Y	N	N/R	N	N	N	N	N/A	N	N	Y	Y	N/A	Y	Y	Y	5	14	0.36
Kim_2021	Y	Y	N/A	Y	N	N	N	N	Y	N	Y	Y	N/A	Y	Y	Y	8	14	0.57
Lai_2019	Y	N	N/A	N	N	N	N	N	Y	N	Y	Y	N/A	Y	N	Y	5	14	0.36
Mears_2020a	Y	Y	N/A	Y	N	N	N	N/A	N	N	Y	Y	N/A	Y	N	N	5	13	0.38
Mears_2020b	Y	Y	N/A	Y	N	N	N	N/A	N	N	N	Y	N/A	Y	N	N	4	13	0.31
Ngom_2016	Y	Y	N/A	Y	N	N	N	N	Y	N	Y	Y	N/A	Y	N	N	6	14	0.43
Sander_2017	Y	Y	N/A	Y	N	N	N	N	Y	N	N	Y	N/A	Y	Y	Y	7	14	0.50
Shen_2017	Y	Y	N/A	Y	N	N	N	N/A	Y	N	Y	Y	N/A	N	N	N	5	13	0.38
Tsai_2016	Y	Y	N/A	Y	N	Y	Y	N/A	N	N	N	Y	N/A	Y	N	N	6	13	0.46
Wang_2019	Y	Y	N/A	Y	N	N	N	N	Y	N	Y	Y	N/A	Y	Y	Y	8	14	0.57
Wheeler_2015	Y	Y	Y	Y	N	Y	Y	N	Y	N	N	Y	N/A	Y	N	N	8	15	0.53
Wu_2017	Y	Y	N/A	Y	N	N	N	N	Y	N	Y	Y	N/A	Y	Y	N	7	14	0.50
Wu_2018	Y	Y	N/A	Y	N	Y	Y	N	Y	N	Y	Y	N/A	Y	Y	Y	10	14	0.71
