# Supplementary material for: Green Space Quality and Health: A Systematic Review
Source: Int J Environ Res Public Health. 2021 Oct 20;18(21):11028. doi: 10.3390/ijerph182111028 (PMC8582763; doi:10.3390/ijerph182111028)
Supplement: Supplementary file 1 [file ijerph-18-11028-s001.zip › ijerph-1381739-supplementary/Green space quality and health - R1 - Supplementary file S3.pdf]

Green space quality and health: a systematic review (PY Nguyen et al, 2021)

Supplementary table S3: List of excluded studies from full text reviews

Citation	Reasons for exclusion
Adams J, Barnett L, Veitch J. What sort of playground design facilitates physical activity and encourages children to use diverse motor skills?. Journal of Science and Medicine in Sport. 2018 Nov 1;21:S12.	Not specific to green space
Astell-Burt T, Feng X. Does sleep grow on trees? A longitudinal study to investigate potential prevention of insufficient sleep with different types of urban green space. SSM-population health. 2020 Apr 1;10:100497.	No health outcomes
Björk J, Albin M, Grahn P, Jacobsson H, Ardö J, Wadbro J, Östergren PO, Skärbäck E. Recreational values of the natural environment in relation to neighbourhood satisfaction, physical activity, obesity and wellbeing. Journal of Epidemiology & Community Health. 2008 Apr 1;62(4):e2-.	No quality measure identified
Browning MH, Lee K, Wolf KL. Tree cover shows an inverse relationship with depressive symptoms in elderly residents living in US nursing homes. Urban Forestry & Urban Greening. 2019 May 1;41:23-32.	No quality measure identified
Cariñanos P, Casares-Porcel M, de la Guardia CD, Aira MJ, Belmonte J, Boi M, Elvira-Rendueles B, De Linares C, Fernández-Rodriguez S, Maya-Manzano JM, Pérez-Badía R. Assessing allergenicity in urban parks: A nature-based solution to reduce the impact on public health. Environmental Research. 2017 May 1;155:219-27.	No health outcomes
Carthy P, Lyons S, Nolan A. Characterising urban green space density and footpath-accessibility in models of BMI. BMC Public Health. 2020 Dec;20:1-2.	No quality measure identified
Cervinka R, Schwab M, Haluza D. Investigating the qualities of a recreational forest: Findings from the cross-sectional hallerwald case study. International journal of environmental research and public health. 2020 Jan;17(5):1676.	No quality measure identified
Coventry, P.A., Neale, C., Dyke, A., Pateman, R. and Cinderby, S._2019. The mental health benefits of purposeful activities in public green spaces in urban and semi-urban neighbourhoods: A mixed-methods pilot and proof of concept study. International journal of environmental research and public health, 16(15), p.2712.	No quality measure identified
Cusack L, Larkin A, Carozza SE, Hystad P. Associations between multiple green space measures and birth weight across two US cities. Health & place. 2017 Sep 1;47:36-43.	No quality measure identified
Dennis M, Cook PA, James P, Wheater CP, Lindley SJ. Relationships between health outcomes in older populations and urban green infrastructure size, quality and proximity. BMC public health. 2020 Dec;20:1-5.	No quality measure identified
Dzhambov A, Hartig T, Markevych I, Tilov B, Dimitrova D. Urban residential greenspace and mental health in youth: Different approaches to testing multiple pathways yield different conclusions. Environmental research. 2018 Jan 1;160:47-59.	No quality measure identified
Engemann K, Svenning JC, Arge L, Brandt J, Erikstrup C, Geels C, Hertel O, Mortensen PB, Plana-Ripoll O, Tsirogiannis C, Sabel CE. Associations between growing up in natural environments and subsequent psychiatric disorders in Denmark. Environmental research. 2020 Sep 1;188:109788.	Not specific to green space
Frank LD, Saelens BE, Chapman J, Sallis JF, Kerr J, Glanz K, Couch SC, Learnihan V, Zhou C, Colburn T, Cain KL. Objective assessment of obesogenic environments in youth: geographic information system methods and spatial findings from the neighborhood impact on kids study. American journal of preventive medicine. 2012 May 1;42(5):e47-55.	Not specific to green space
Gong Y, Gallacher J, Palmer S, Fone D. Neighbourhood green space, physical function and participation in physical activities among elderly men: the Caerphilly Prospective study. International Journal of Behavioral Nutrition and Physical Activity. 2014 Dec;11(1):1-1.	No quality measure identified
Grahn P, Stigsdotter UK. The relation between perceived sensory dimensions of urban green space and stress restoration. Landscape and urban planning. 2010 Mar 15;94(3-4):264-75.	No quality measure identified
Gražulevičienė R, Andrušaitytė S, Dėdelė A, Gražulevičius T, Valius L, Kapustinskienė V, Bendokienė I. Environmental quality perceptions and health: A cross-sectional study of citizens of Kaunas, Lithuania. International journal of environmental research and public health. 2020 Jan;17(12):4420.	Not specific to green space
Hassink J, Vaandrager L, Buist Y, De Bruin S. Characteristics and challenges for the development of nature-based adult day services in urban areas for people with dementia and their family caregivers. International journal of environmental research and public health. 2019 Jan;16(8):1337.	No health outcomes
Hedblom M, Gunnarsson B, Iravani B, Knez I, Schaefer M, Thorsson P, Lundström JN. Reduction of physiological stress by urban green space in a multisensory virtual experiment. Scientific reports. 2019 Jul 12;9(1):1-1.	Digitalised simulation of green space

Hedblom M, Gunnarsson B, Schaefer M, Knez I, Thorsson P, Lundström JN. Sounds of nature in the city: no evidence of bird song improving stress recovery. International journal of environmental research and public health. 2019 Jan;16(8):1390.	Digitalised simulation of green space
Hipp JA, Ogunseitan OA. Effect of environmental conditions on perceived psychological restorativeness of coastal parks. Journal of Environmental Psychology. 2011 Dec 1;31(4):421-9.	Not specific to green space
Jimenez MP, Wellenius GA, James P, Subramanian SV, Buka S, Eaton C, Gilman SE, Loucks EB. Associations of types of green space across the life-course with blood pressure and body mass index. Environmental research. 2020 Jun 1;185:109411.	No quality measure identified
Lanza K, Alcazar M, Hoelscher DM, Kohl HW. Effects of trees, gardens, and nature trails on heat index and child health: design and methods of the Green Schoolyards Project. BMC public health. 2021 Dec;21(1):1-2.	No health outcomes
Larson LR, Jennings V, Cloutier SA. Public parks and wellbeing in urban areas of the United States. PLoS one. 2016 Apr 7;11(4):e0153211.	No quality measure identified
Li Z, Zeng R, Ye M. Investigation of the relationship between place characteristics and child behavior in residential landscape spaces: a case study on the Century Sunshine Garden Residential Quarter in Hefei. Frontiers of Architectural Research. 2012 Jun 1;1(2):186-95.	No health outcomes
Lovasi GS, Quinn JW, Neckerman KM, Perzanowski MS, Rundle A. Children living in areas with more street trees have lower prevalence of asthma. Journal of Epidemiology & Community Health. 2008 Jul 1;62(7):647-9.	No quality measure identified
Mason KE, Pearce N, Cummins S. Do neighbourhood characteristics act together to influence BMI? A cross-sectional study of urban parks and takeaway/fast-food stores as modifiers of the effect of physical activity facilities. Social Science & Medicine. 2020 Sep 1;261:113242.	No quality measure identified
Mazumdar S, Chong S, Astell-Burt T, Feng X, Morgan G, Jalaludin B. Which Green Space Metric Best Predicts a Lowered Odds of Type 2 Diabetes?. International journal of environmental research and public health. 2021 Jan;18(8):4088.	No quality measure identified
Miralles-Guasch C, Dopico J, Delclòs-Alió X, Knobel P, Marquet O, Maneja-Zaragoza R, Schipperijn J, Vich G. Natural landscape, infrastructure, and health: The physical activity implications of urban green space composition among the elderly. International journal of environmental research and public health. 2019 Jan;16(20):3986.	No health outcomes
Moreira TC, Polizel JL, Santos ID, Silva Filho DF, Bensenor I, Lotufo PA, Mauad T. Green spaces, land cover, street trees and hypertension in the megacity of São Paulo. International journal of environmental research and public health. 2020 Jan;17(3):725.	No quality measure identified
Nath TK, Han SS, Lechner AM. Urban green space and well-being in Kuala Lumpur, Malaysia. Urban Forestry & Urban Greening. 2018 Dec 1;36:34-41.	No quality measure identified
Nordh H, Østby K. Pocket parks for people–A study of park design and use. Urban forestry & urban greening. 2013 Jan 1;12(1):12-7.	No quality measure identified
Paddle E, Gilliland J. Orange is the new green: Exploring the restorative capacity of seasonal foliage in schoolyard trees. International journal of environmental research and public health. 2016 May;13(5):497.	Digitalised simulation of green space
Parra DC, Gomez LF, Sarmiento OL, Buchner D, Brownson R, Schimd T, Gomez V, Lobelo F. Perceived and objective neighborhood environment attributes and health related quality of life among the elderly in Bogota, Colombia. Social science & medicine. 2010 Apr 1;70(7):1070-6.	Not specific to green space
Potwarka LR, Kaczynski AT, Flack AL. Places to play: association of park space and facilities with healthy weight status among children. Journal of community health. 2008 Oct 1;33(5):344-50.	Not specific to green space
Pugh TA, MacKenzie AR, Whyatt JD, Hewitt CN. Effectiveness of green infrastructure for improvement of air quality in urban street canyons. Environmental science & technology. 2012 Jul 17;46(14):7692-9.	No quality measure identified
Puhakka R, Rantala O, Roslund MI, Rajaniemi J, Laitinen OH, Sinkkonen A, ADELE Research Group. Greening of daycare yards with biodiverse materials affords well-being, play and environmental relationships. International journal of environmental research and public health. 2019 Jan;16(16):2948.	No health outcomes
Qiao Y, Chen Z, Zheng T. Deciphering the Link Between Mental Health and Green Space in Shenzhen, China: The Mediating Impact of Residents' Satisfaction. Frontiers in Public Health. 2021;9:52.	No quality measure identified
Schinasi LH, Quick H, Clougherty JE, De Roos AJ. Greenspace and infant mortality in Philadelphia, PA. Journal of Urban Health. 2019 Jun;96(3):497-506.	No quality measure identified
Song S, Yap W, Hou Y, Yuen B. Neighbourhood built Environment, physical activity, and physical health among older adults in Singapore: A simultaneous equations approach. Journal of Transport & Health. 2020 Sep 1;18:100881.	Not specific to green space
South EC, Hohl BC, Kondo MC, MacDonald JM, Branas CC. Effect of greening vacant land on mental health of community-dwelling adults: a cluster randomized trial. JAMA network open. 2018 Jul 6;1(3):e180298-.	Not specific to green space

Van den Berg AE, Jorgensen A, Wilson ER. Evaluating restoration in urban green spaces: Does setting type make a difference?. Landscape and Urban Planning. 2014 Jul 1;127:173-81.	Digitalised simulation of green space
Van Herzele A, de Vries S. Linking green space to health: A comparative study of two urban neighbourhoods in Ghent, Belgium. Population and Environment. 2012 Dec 1;34(2):171-93.	No quality measure identified
Wang R, Helbich M, Yao Y, Zhang J, Liu P, Yuan Y, Liu Y. Urban greenery and mental wellbeing in adults: Cross-sectional mediation analyses on multiple pathways across different greenery measures. Environmental research. 2019 Sep 1;176:108535.	No quality measure identified
Wang X, Rodiek S, Wu C, Chen Y, Li Y. Stress recovery and restorative effects of viewing different urban park scenes in Shanghai, China. Urban forestry & urban greening. 2016 Jan 1;15:112-22.	Digitalised simulation of green space
Warembourg C, Nieuwenhuijsen M, Ballester F, de Castro M, Chatzi L, Esplugues A, Heude B, Maitre L, McEachan R, Robinson O, Slama R. Urban environment during early-life and blood pressure in children. InISEE Conference Abstracts 2020 Oct 26 (Vol. 2020, No. 1).	No quality measure identified
Younan D, Tuvblad C, Li L, Wu J, Lurmann F, Franklin M, Berhane K, McConnell R, Wu AH, Baker LA, Chen JC. Environmental determinants of aggression in adolescents: Role of urban neighborhood green space. Journal of the American academy of child & adolescent psychiatry. 2016 Jul 1;55(7):591-601.	No quality measure identified
Yuen JW, Chang KK, Wong FK, Wong FY, Siu JY, Ho HC, Wong MS, Ho J, Chan KL, Yang L. Influence of urban green space and facility accessibility on exercise and healthy diet in Hong Kong. International journal of environmental research and public health. 2019 Jan;16(9):1514.	No health outcomes
Zenk SN, Tarlov E, Wing C, Slater S, Jones KK, Fitzgibbon M, Powell LM. Does the built environment influence the effectiveness of behavioral weight management interventions?. Preventive medicine. 2019 Sep 1;126:105776.	No quality measure identified
Zhao J, Wu J, Wang H. Characteristics of urban streets in relation to perceived restorativeness. Journal of exposure science & environmental epidemiology. 2020 Mar;30(2):309-19.	Digitalised simulation of green space
Zhao W, Li H, Zhu X, Ge T. Effect of birdsong soundscape on perceived restorativeness in an urban park. International Journal of Environmental Research and Public Health. 2020 Jan;17(16):5659.	Digitalised simulation of green space
