# Supplementary material for: Green Space Quality and Health: A Systematic Review
Source: Int J Environ Res Public Health. 2021 Oct 20;18(21):11028. doi: 10.3390/ijerph182111028 (PMC8582763; doi:10.3390/ijerph182111028)
Supplement: Supplementary file 1 [file ijerph-18-11028-s001.zip › ijerph-1381739-supplementary/Green space quality and health - R1 - Supplementary file S4.pdf]

Green space quality and health: a systematic review (PY Nguyen et al, 2021)

Supplementary File S4. Summary of findings (expanded)

Study*	Measure of quality	Tool(s) used to assess gren space quality**	Outcome	Tool(s) used to assess outcome **	Findings from study***	Direction of effect≠
**Environment / land cover type (n=22)**						
Marselle_2015 (n=127) [19]	Environment types: natural and semi-natural places, green corridor, urban green space, farmland, urban public spaces, coastal, mixture	Self-reported	Positive & negative affect	PANAS scale	Environmental types of an outdoor walk did not predict post-walk emotional states of participants.	(o)
Stas_2021 (n=189) [20]	Vegetation species & cover types: trees vs grass	GIS analysis (1km buffer)	Severe tree pollen allergy event	Self-reported	Higher grass coverage were protective for severe allergy (OR=0.655, 95% CI 0.446–0.960), but only on the day of the severe allergy event. No significant association was observed for gardens (OR=0.987, 95% CI 0.706-1.380) or forests (OR=0.748, 95% CI 0.521-1.074). Density of Alnus species were protective for severe allergy (OR=0.622, 95% CI 0.411–0.942), but not Betula (OR=1.384, 95% CI 0.750-2.554) or Corylus species (OR=0.806, 95% CI 0.447–1.454), after adjusting for birch pollen and air pollution.	(+)
Astell-Burt_2020a (n=109,688) [21]	Vegetation cover types: trees vs grass	GIS analysis (1.6km buffer)	Dementia: first medication prescription, first hospitalisation and deaths	Medical records	High tree coverage (≥30% vs <10%) was associated with lower rates of dementia detected using hospital and death records (IHR = 1.33, 95%CI 1.07-1.66). Anti-dementia medication-based detection also indicated lower dementia risk with more open grass (≥20% vs <5%) (IHR = 0.83, 95%CI 0.67-1.03).	(+)
Astell-Burt_2019 (n=46,786) [22]	Vegetation cover types: trees, grass vs low-lying vegetation	GIS analysis (1.6km buffer)	Psychological stress; depression/anxiety; general health	K10-PDS; self-reported	High tree coverage was associated with lower incidence of psychological distress (OR=0.69; 95% CI 0.54-0.88) and poor general health (OR=0.67; 95% CI 0.57-0.80) while the opposite was observed for grass coverage (OR=1.71; 95% CI 1.25-2.28 for psychological distress and OR=1.47; 95% CI 1.12-1.91 for poor general health). High coverage of low-lying vegetation was not consistently associated with either outcome (OR from 0.88 to 1.11, p>0.05). No green space indicator was associated with depression or anxiety (OR from 0.86 to 1.13, p>0.05).	(+)
Richardson_2018 (n=46,093) [23]	Natural space types: parks, woods, open waters	GIS analysis (100m)	Live births	Medical records	Birth weight was modestly associated with the total amount of natural space around the mother's home, but a significant association was not observed for specific environment types such as parks (β=-0.38, 95% CI 0.87-0.10, woodlands: (β=1.46, 95% CI 0.21-3.13) or open waters: (β=1.04, 95% CI 1.24-3.32).	(+)
Astell-Burt_2020c (n=45,644) [24]	Vegetation cover types: trees vs grass	GIS analysis (1.6km buffer)	Memory complaints; self-rated memory	Semantic differential scale	No significant associations were observed between any vegetation coverage type and fewer subjective memory complaints or better self-rated memory. Multilevel analyses of 8 latent classes showed that when participants had greater more tree coverage relative to open grass and shrubs, more favourable results were observed for both memory-outcomes.	(o)

Study*	Measure of quality	Tool(s) used to assess gren space quality**	Outcome	Tool(s) used to assess outcome **	Findings from study***	Direction of effect≠
Astell-Burt_2021 (n=45,644)	Vegetation cover types: trees vs open grass	GIS analysis (1.6km buffer)	CVD mortality, CVD events, AMI	Medical records	Higher tree coverage tended to be associated with lower CVD mortality (HR=0.75, 95% CI 0.47-1.16), lower fatal or non-fatal CVD events (HR=0.92, 95% CI 0.77-1.11), and lower AMI risk (HR=0.77, 95% CI 0.42-1.36). Higher open grass coverage tended to be associated with lower all-cause mortality (HR=0.73, 95% CI 0.45-1.12), lower CVD mortality (HR=0.76, 95% CI 0.38-1.36) and lower fatal or non-fatal CVD events (HR=0.82, 95% CI 0.63-1.05), but higher AMI risk (HR=1.17, 95% CI 0.53-2.40).	(o)
Gernes_2019 (n=478) [25]	Land cover diversity	GIS analysis (100m, 400m and 800m buffer)	Outdoor allergen sensitisation; allergic rhinitis	Skin prick tests; clinically diagnosed	A 10% increase in grass coverage was associated with an increased risk of sensitisation to grass pollens at both 400m and 800m buffers (OR=1.27, 95% CI 1.02-1.58 and OR=1.27; 95% CI 1.01−1.60, respectively). No significant association was found between tree coverage and tree pollen sensitization (OR from = 0.86−0.96, p>0.05). Although not statistically significant, ORs consistent in magnitude were detected for allergic rhinitis per 10% in grass coverage (OR = 0.92; 95% CI 0.75-1.12 at 400m) and tree coverage (OR=0.99, 95% CI 0.86-1.14 at 400m).	(–)
Donovan_2018 (n=39,108) [26]	Vegetation cover types	GIS analysis (225m buffer)	Childhood asthma	Medical records	A higher diversity in natural land cover types in the neighbourhood was associated a lower risk of childhood asthma (OR=0.93, 95% CI 0.89–0.99). Certain land cover types, such as exotic conifers or gorse (Ulex europaeus), were associated with an increased risk of asthma (OR=1.04, 95% CI 1.01-1.08 and OR=1.03, 95% CI=1.00–1.06, respectively).	(+)(–)
Parmes_2020 (n=8,063) [27]	Forest types: deciduous, coniferous vs mixed	GIS analysis (500m buffer)	Wheezing, asthma, allergic rhinitis, eczema	Parental reported	Children living near coniferous forests had significantly greater odds of reporting wheezing (OR=1.76, 95% CI 1.05-2.97), asthma (OR=2.54, 95% CI 1.11-5.82) and allergic rhinitis (OR 3.40, 95% CI 1.83-6.30). No significant associations were found for broad-leaf or mixed forests (OR from 0.53 to 1.09, p>0.05).	(–)
Jarvis_2020 (n=1,960,575) [28]	Land cover types: coniferous, deciduous, shrub, grass-herbs, water, buildings, paved surfaces	GIS analysis (250m, 500m and 1,000m buffers)	General health, mental health, common mental disorders	Semantic differential scale	Lower risks of poor mental health and common mental disorders were associated with exposure to shrubs within 250m radial buffer (OR=0.741, 95% CI=0.576-0.953). There was no significant association on both general health and mental health by grass-herbs vegetation (OR from 0.980 to 1.009, p>0.05), coniferous trees (OR from 0.970 to 1.010, p>0.05) or deciduous trees (OR from 0.989 to 1.002, p>0.05).	(+)
Nishigaki_2020 (n=126,878) [29]	Vegetation cover types: trees vs grass	GIS analysis (300m-3km buffer)	Depression	SGD	In urban areas, high tree coverage correlated with lower odds of depression (OR 0.94, 95% CI 0.89-1.00). In rural areas, moderate amounts of grassland were associated with lower odds of depression (OR 0.91, 95% CI 0.83-1.00).	(+)
Wyles_2019 (n=4,515) [30]	Environment types:<br>* Coastal: in a seaside resort or town, other seaside coastline (including beaches and cliffs)<br>* Rural green: the countryside (including areas around towns / cities)<br>* Urban green: in a town or city	Self-reported	Restorativeness	Semantic differential scale	Visitors recalled greater restoration following visits to coastal locations (β= 0.14, SE 0.04) than urban green space. No significant difference was observed for rural green spaces (β=0.05, SE 0.03).	(+)
Reid_2017 (n=1,387) [31]	Vegetation cover types: trees vs grass	GIS analysis (300m and 1,000m buffer)	Perceived health	Semantic differential scale	At 1000m radial buffer, tree density had a dose-dependent positive effect on self-reported health rating (RR=1.36, 95% CI 1.13-1.64), but grass density did not (RR=1.21, 95% CI 0.96-1.53).	(+)

Study*	Measure of quality	Tool(s) used to assess gren space quality**	Outcome	Tool(s) used to assess outcome **	Findings from study***	Direction of effect≠
Jiang_2020 (n=212) [32]	Vegetation cover types: trees vs low-lying vegetation	GIS analysis (400m, 800m, 1600m buffer)	General health; stress level	SF-12; PSS	At 800m radial buffer, higher coverage tree canopy around home was related to better overall health (β=3.56, SE 1.52) and an increased capacity to control stress (β=0.84, SE 0.38). Higher coverage of understory vegetation did not improve health (β=-0.39, SE 3.80) and was associated with increased overall stress (β=-3.45, SE 1.65).	(+)(–)
Egorov_2020 (n=186) [33]	Vegetation cover types: trees vs grass	GIS analysis (500m buffer)	Allostatic load	Clinically measured	High tree coverage was associated with reduction in allostatic load of residents (OR=0.86, 95% CI 0.78-0.93). Grass cover was inversely correlated with tree cover and was not associated with reduced allostatic load (OR=1.04, 95% CI 0.96-1.14).	(+)
Wheeler_2015 (n=31,672 LSOAs) [34]	Land cover diversity; Environment types: broadleaf woodlands, coniferous woodlands, semi-natural grassland, improved grassland, arable and horticulture, mountain-heath-bog, saltwater, freshwater and coastal	SDI; GIS analysis (100m buffer)	Health status	Semantic differential scale	High prevalence of good health was associated with land cover diversity (β=0.27, 95% CI 0.22-0.33). Positive associations were demonstrated with these environment types: broadleaf woodlands (β=0.032, 95% CI 0.029-0.035), arable and horticulture (β=0.004, 95% CI 0.002-0.005), improved grasslands (β=0.016, 95% CI 0.01-0.018), saltwater (β=0.074, 95% CI 0.032, 0.117) and coastal environment (β=0.019, 95% CI 0.010-0.027). Other environments demonstrate non-significant positive change in crude models, but their association was attenuated by adjustment for deprivation indices and urban/rural categories (β from -0.004 to 0.006, p>0.05).	(+)
Aerts_2020 (n=1,872 census tracts) [35]	Land cover types: gardens, forests vs grassland	GIS analysis	Respiratory diseases	Medication sales	Positive associations were observed between medication sales for obstructive airway diseases (OAD) and coverage of grassland (β=0.098, 95% CI 0.061-0.135 to β=0.138, 95% CI 0.049-0.227) and gardens (β=0.071, 95% CI 0.047-0.095 to β=0.120, 95% CI 0.096-0.144) across both sexes and age groups. There was no association between OAD medication sales and forest cover (β=-0.012 to 0.010, p>0.05).	(+)
Dennis_2020 (n=1,673 LSOAs) [36]	Land cover diversity; vegetation cover types (ground, canopy vs field-level)	SDI; GIS analysis (100m, 200m and 300m buffer)	Chronic morbidity prevalence	CIDR	Land cover diversity exhibited consistent association with lower chronic morbidities among high-income, older neighbourhoods (β=-0.116, p<0.001 and β=0.084, p<0.01, respectively). Similar benefits were found for all three informal vegetation cover types: ground (β=-0.099, p<0.01), canopy (β=-0.130, p<0.01) and field level vegetation (β=-0.070, p<0.05), but not for young and mid-aged neighbourhoods.	(+)
Sander_2017 (n=546 census blocks) [37]	Land cover types: water, forest, canopy, impervious surfaces, and grass	GIS analysis	BMI	Self-measured height & weight	Young-adult males exhibited the only significant relationships with canopy (β=0.167, p<0.01), forest (β=−0.011, p<0.01), and grass (β=0.0058, p<0.05). Green space impervious surface was significantly and negatively related to BMI for middle-adult females (β=−0.0082, p<0.05) and males (β =−0.0052, p<0.05). Water in green spaces exhibited significant, negative relationships to BMI for older-adult females (β=−0.045, p<0.05) and middle-adult males (β=−0.040, p<0.01)	(+)
Wu_2017 (n=543 districts) [38]	Vegetation cover types: forest, grassland, average tree canopy and near-road tree canopy	GIS analysis (50m and 100m buffers)	Autism	Medical records	Higher coverage of forest (RR=0.90, 95% CI 0.84-0.95), grassland (RR=0.90, 95% CI: 0.83-0.97), tree canopy (RR=0.89, 95% CI: 0.83-0.95) and specifically near-road tree canopy (RR=0.81, 95% CI 0.73-0.91) was associated with lower prevalence of autism.	(+)
Wu_2018 (n=187 census tracts) [39]	Land cover types: water, open land, developed land, barren land, forest, shrub land, grassland, agriculture and wetland	GIS analysis (50m and 100m buffers)	Sudden unexpected deaths	Medical records	SUD incidence was inversely associated with greenway density (RR=0.82, 95% CI 0.69-0.97) and forest density (RR=0.90, 95% CI 0.81-0.99), but not grassland (RR=0.91, 95% CI 0.65-1.29) or tree canopy (RR=0.95, 95% CI 0.86-1.04).	(+)
**Natural features (n=15)**						

Study*	Measure of quality	Tool(s) used to assess gren space quality**	Outcome	Tool(s) used to assess outcome **	Findings from study***	Direction of effect≠
Marselle_2015 (n=127) [19]	Perceived naturalness; bird, butterfly, plants & trees biodiversity	Semantic differential scale, manual counting of species	Positive & negative affect	PANAS scale	Bird diversity (β=0.444, SE 0.144) and the environment's perceived naturalness (β=0.020, SE 0.042) was associated with negative emotions after an outdoor walk, and this negative effect was moderated by restorative quality. Butterfly, plants & trees diversity did not significantly improve emotional wellbeing.	(–)
Astell-Burt_2020b (n=46,786) [40]	Tree coverage	GIS analysis (1.6km buffer)	Diabetes, hypertension and cardiovascular diseases	Medical records	Lower odds of incident diabetes (OR=0.988, 95% CI 0.981-0.994), hypertension (OR=0.993, 95% CI 0.989-0.997) and CVD (OR=0.993, 95% CI 0.988-0.998) were associated with a 1% increase in tree canopy, but not total green space.	(+)
Wyles_2019 (n=4,515) [30]	Protected/designated area status	Assigned by national agency	Restorativeness	Semantic differential scale	Visitors recalled greater restoration following visits to sites of higher environmental quality (operationalized by protected/designated area status, for example, nature reserves) (β=0.08, SE 0.06).	(+)
Leng_2020 (n=4,155) [41]	Presence of evergreen trees	Environmental audits	Obesity, hypertension, diabetes, dyslipidaemia, stroke risk	Clinically measured BMI, blood pressure, blood glucose & lipid tests, stroke risk score card	In a winter city, a significant relationship was detected between presence of evergreen trees in green spaces and being overweight/obese (OR=1.44, 95% CI 1.09-1.91) and hypertension (OR=0.41, 95% CI 0.19-0.90). Non-significant association was found with diabetes (OR=1.85, 95% CI 0.77-4.43), dyslipidaemia (OR=1.06, 95% CI 0.54-2.11) or stroke risk (OR=0.90, 95% CI 0.62-1.32).	(+)
Camargo_2017 (n=1,392) [42]	Conditions of trees	Semantic differential scale	Quality of life	EUROHIS-QOL	Better tree conditions were associated with better quality of life among park visitors (β=1.20, 95% CI 1.07-1.34)	(+)
Zhang_2019b (n=909) [43]	Tree density	POST	Quality of life	WHOQOL-BREF	Tree density in park was positively associated with social aspect in elderly residents (β=0.132, 95% CI 0.003-0.261), but not with other aspects of quality of life (physical, psychological or environmental) (β=0.074, 0.053 and -0.018 respectively, p>0.1).	(+)
Carter_2014 (n=440) [44]	Retention of green space & bushland:<br>* Green area not disappearing<br>* Access to enough bushland<br>* Access to bushland area within neighbourhood	Semantic differential scale	Physical function	SF-36v2	Retention of green space and bushland did not significantly improve physical health of residents (OR=1.82, p=0.097).	(o)
Tan_2019 (n=326) [45]	Tree density	Environmental audits	Physical functioning, physical role, bodily pain and emotional role	SF-12v2	Tree density in urban green space had no significant association with physical or emotional health of residents in both Taiwan and Hong Kong (rs from -0.101 to 0.109, p>0.05).	(o)
Pazhouhanfar_2018 (n=250) [46]	Tree and greening, flowers, sun, water, fresh air, and bird voice	Semantic differential scale	Mood ratings (relaxed / happy / excited / calmed)	Semantic differential scale	Flowers (β=0.19, p=0.02), tree and greening (β=0.21, p=0.01) and fresh air (β=0.31, p=0.01) predicted positive mood states among park visitors; while sun (β=-0.13, p=0.04) negatively affected mood states.	(+)
Zhu_2020 (n=240) [47]	Sky index, soft/hard surface ratio, vertical vegetation coverage	Grid pixel calculation	Restorative effect	PRS	Sky index of a park's landscape correlated with its perceived restorative effect (rs=-0.611, p<0.01). Soft/hard surface ratio (rs=0.539, p<0.01) and vertical vegetation coverage (rs=0.613, p<0.01) was negatively correlated with its restorative effect.	(+)(–)

Study*	Measure of quality	Tool(s) used to assess gren space quality**	Outcome	Tool(s) used to assess outcome **	Findings from study***	Direction of effect≠
					Correlation was largest with tree coverage, followed by herbaceous vegetation and shrubs (rs=0.679, 0.596 and 0.522 respectively, p<0.01).	
Wood_2018 (n=128) [48]	Ecological study richness score:<br>* Plant diversity<br>* Bird diversity<br>* Bee/butterfly diversity<br>* Habitat number	Environmental audits; SDI	Restorative effect	Modified ART	Ecological richness score explained 43% of the variance in restorative benefits between parks (β=0.485, SE 0.149).	(+)
Honold_2016 (n=32) [49]	Diversity of vegetation<br>* Façade<br>* Designs (shapes, colours etc.)<br>* Building shapes<br>* Vanishing points<br>* Angles	Semantic differential scale	Stress level	Hair cortisol level (immunoassay)	Quantity or diversity of neighbourhood vegetation alone did not predict stress level (β=-0.10 and 0.28 respectively, p>0.05), but their interaction term has a significant effect (β=-0.40, p<0.05). Residents' stress level were lowest when their window view was characterised by high vegetation coverage and diversity, but was highest when the view featured high coverage, low diversity vegetation.	(o)
Wheeler_2015 (n=31,672 LSOAs) [34]	Bird species richness, freshwater quality indicator, density of protected area density	Bird occurrence atlas, routine surface water testing	Health status	Semantic differential scale	High prevalence of good health was associated with bird species richness (β=0.02, 95% CI 0.001-0.04) and high density of protected areas (β=0.13, 95% CI 0.11-0.15). Freshwater quality indicator was negatively associated with good health (β=-0.10, 95% CI -0.15 to -0.04)	(+)
Mears_2020b (n=345 LSOAs) [50]	Bird biodiversity	Citizen science programme data	Poor general health	Semantic differential scale	LSOAs with higher bird diversity did not have significantly better general health (β=0.273, SE 0.188)	(o)
Lai_2019 (n=174 zip codes) [51]	Pollen allergenicity of trees	Street tree census	Asthma prevalence	Medical records	Allergenicity of street trees was correlated with local asthma hospitalisation rates (β=0.02, p<0.05).	(–)
**Infrastructure & amenities (n=14)**						
Droomers_2015 (n=48,132) [52]	Green intervention projects:<br>* Replacing vacant land with parks<br>* Added areas for playing and recreation<br>* Better paths and tracks<br>* Improved drainage<br>* Landscaping<br>* Maintenance	Construction & installation of new amenities	Health status	Semantic differential scale	The deprived neighbourhoods that intervened in green space did not show more favourable changes in good general health compared to all the different groups of control areas (t=−0.03, 95% CI −0.17 to 0.11)	(o)
Dobbinson_2020 (n=1,670) [53]	Refurbishments to existing amenities:<br>* Playground equipment<br>* Quality walking paths<br>* Shade & shade-sail	Construction & installation of new amenities	Positive & negative affect	PANAS scale	Refurbishments did not have significant association with the emotional states of park visitors. Change in positive affect by 1-year and 2-year post-intervention was −3.22 (p=0.23) and −3.90 (p=0.20) respectively. Change in negative affect by 1-year and 2-year post-intervention was −0.75 (p=0.70) and −3.14 (p=0.28) respectively.	(o)
Wood_2017 (n=492) [54]	Park functions	POSDAT	Mental wellbeing	WEMWBS	Positive mental health was not only associated with parks with a nature focus (β=0.11, SE 0.05), but also with green spaces providing facilities for recreational (β=0.11, SE 0.05) and sporting activities (β=0.43, SE 0.21).	(+)

Study*	Measure of quality	Tool(s) used to assess gren space quality**	Outcome	Tool(s) used to assess outcome **	Findings from study***	Direction of effect≠
McCarthy_2017 (n=13,469) [55]	Playground quality: useability, cleanliness and maintenance, distinct areas for different age groups, colourful equipment (3+ colours), shade cover for 25%+ area, benches, fence for 50%+ area, separation from roads	Environmental audits	BMI	Clinically measured	There was no significant association between quality of playgrounds in parks and weight status among youths (OR=0.82, 95% CI 0.66-1.03).	(o)
Rundle_2013 (n=13,102) [56]	Number of recreational facilities	Environmental audits	BMI	Clinically measured	The number of recreational facilities was not significantly associated with BMI (β=0.27, 95% CI −0.12 to 0.66), nor was diversity in recreational facility types (β=0.20, 95% CI −0.20 to 0.60).	(o)
Bojorquez_2018 (n=2,345) [57]b	Park quality score: bathrooms, lighting, playground, etc. (9 items in total)	Environmental audits	Depressive symptoms	CES-D	Park quality score was not associated with lower depressive symptoms among the resident population for both 400m (β=-0.02, 95% CI –0.04 to 0.01) and 800m (β=0.00, 95% CI –0.02 to 0.02) radial buffer.	(o)
Camargo_2017 (n=1,392) [42]	Walking paths conditions	Semantic differential scale	Quality of life	EUROHIS-QOL	Better walking paths' conditions significantly improved quality of life of park visitors (β=1.21, 95% CI 1.03-1.41).	(+)
Zhang_2019b (n=909) [43]	Amenities:<br>* Children’s play equipment<br>* Seating facilities<br>* Dog litter bags<br>* Water sources for dogs<br>* Drinking fountains<br>* Parking facilities<br>* Public transport<br>Variety of activities allowed	POST	Quality of life	WHOQOL-BREF	Park amenities had no significant association with all aspects of quality of life (physical, psychological, social or environmental) in elderly residents (β=-0.049, 0.006, 0.043 and 0.006 respectively, p>0.1). Similarly, having a variety of activities within parks had no impact on quality of life (β=-0.031, -0.018, -0.096 and -0.044 respectively, p>0.1).	(o)
Bai_2013 (n=893) [58]	Availability of facilities of interest	Semantic differential scale	BMI	Self-measured height & weight	Availability of facilities of interest in parks was not significantly associated with BMI (OR=1.00, 95% CI 0.83, 1.22), nor was perceived maintenance of parks (OR=0.90, 95% CI 0.68, 1.20).	(o)
Pope_2018 (n=578) [59]	Maintenance	Dichotomous survey question	Psychological distress	GHQ-12	Maintenance of green space was not significantly associated with reduced psychological distress (OR=0.98, 95% CI 0.63-1.54).	(o)
Tan_2019 (n=326) [45]	Number of facilities and seats	Environmental audits	Physical functioning, physical role, bodily pain and emotional role	SF-12v2	Number of facilities and seats in urban green space had no significant impact on physical or emotional health of residents in both Taiwan and Hong Kong (rs from -0.107 to 0.183, p>0.05).	(o)
Sugiyama_2009 (n=271) [60]	Quality of access paths	Semantic differential scale	Health status<br>Quality of life	No. of days with poor physical/mental health<br>SWLS	Quality of access paths to neighbourhood open space had no significant impact on life satisfaction (OR=1.38, 95% CI 0.73-2.62) nor physical health (OR=1.02, 95% CI 0.52-2.01) of elderly visitors.	(o)

Study*	Measure of quality	Tool(s) used to assess gren space quality**	Outcome	Tool(s) used to assess outcome **	Findings from study***	Direction of effect≠
Mears_2020a (n=345 LSOAs) [61]	Play facilities<br>* Playgrounds<br>* Games areas<br>* Skate or bike parks	Environmental audits	BMI	Clinically measured	LSOAs with more public green spaces designed for children and adolescents did not have significantly lower obesity rates (β=0.004, SE 0.106).	(o)
Ngom_2016 (n=N/A) [62]	Green space functions	GIS databases	Coronary heart disease, cerebrovascular disease, heart failure, diabetes, hypertension	Medical records	Green spaces with sport facilities showed a significant benefit: the most distant population had higher risks of cerebrovascular diseases (pRR=1.11, 95% 1.01-1.22) and diabetes (pRR=1.09, 95% CI=1.03-1.13) than the nearest population. The relationships were not significant for other green space types.	(+)
			**Size (n=11)**			
Wood_2017 (n=492) [54]	Park size	GIS analysis (1.6km buffer)	Mental wellbeing	WEMWBS	The increases in numbers of small parks (<0.4 ha: β=0.20, SE 0.10), district parks (5-20 ha: β=0.65, SE 0.28) and regional parks (20+ ha: β=2.90, SE 1.04) were each positively associated with mental wellbeing, but not the mid-sized local (0.4-1 ha: β=0.05, SE 0.10) and neighbourhood open spaces (1-5 ha: β=0.15, SE 0.12). The effect was much greater for larger regional and district parks than for small parks.	(+)
Stark_2014 (n=44,282) [63]	Park size	GIS analysis (805m buffer)	BMI	Self-measured height & weight	The proportion of neighbourhood dedicated to park space was associated with lower BMI, and the association was observed for both large parks (>6 acres, β=-0.20, 95% CI -0.32 to -0.08) and small parks (£6 acres, β=-0.21, 95% CI -0.31 to -0.10)	(+)
Rundle_2013 (n=13,102) [56]	Park size	GIS analysis (805m buffer)	BMI	Clinically measured	The proportion of neighbourhood dedicated to park space was associated with lower BMI, but the association was only significant for large parks (>6 acres, β=-1.69, 95% CI -2.76 to -0.63)	(+)
Zhang_2019b (n=909) [43]	Park area	GIS analysis (400m and 800m buffers)	Quality of life	WHOQOL-BREF	Park area did not have significant impact on all aspects of quality of life (physical, psychological, social or environmental) in elderly residents (β=0.000, 0.000, -0.004 and 0.000 respectively, p>0.1).	(o)
Tan_2019 (n=326) [45]	Area	Environmental audits	Physical functioning, physical role, bodily pain and emotional role	SF-12v2	Among older adults, size of urban green space has some beneficial effect on reducing physical limitations (rs=0.213, p<0.05) but not for other aspects of health (rs from -0.06 to -0.152, p>0.05).	(+)
Kim_2016 (n=92) [64]	Size of tree canopy	GIS analysis (805m buffer)	Quality of life	PedsQL	Hispanic children living a neighbourhood with larger sizes of tree patches had better quality of life (β=0.357, p=0.023)	(+)
Kim_2014 (n=61) [65]	Size of tree canopy	GIS analysis (805m buffer)	BMI	Clinically measured	Hispanic children living a neighbourhood with larger sizes of tree patches had lower BMI (β=-0.042, p=0.025)	(+)
Dennis_2020 (n=1,673 LSOAs) [36]	Mean patch size	GIS databases	Chronic morbidity prevalence	CIDR	Patch size exhibited association with lower chronic morbidities among high-income, older neighbourhoods (β=− 0.116, p<0.001) and medium-income, younger neighbourhoods (β=0.239, p<0.01, respectively).	(+)

Study*	Measure of quality	Tool(s) used to assess gren space quality**	Outcome	Tool(s) used to assess outcome **	Findings from study***	Direction of effect≠
Wang_2019 (n=369 census tracts) [66]	Patch area	GIS analysis (805m buffer)	All-cause, cardiovascular, chronic respiratory and neoplasm mortality	Medical records	Census tracts with larger green spaces had a lower risk of all-cause (β=-0.000019, p=0.0088) and heart disease mortality (β=-0.000034, p=0.0071)	(+)
Mears_2020a (n=345 LSOAs) [61]	Garden size	GIS analysis (300m buffer)	Obesity rate	Clinically measured BMI	LSOAs with a larger average garden size did not have significantly lower obesity rate among year-6 students (β= -0.550, SE 0.574).	(o)
Mears_2020b (n=345 LSOAs) [50]	Garden size	GIS analysis (300m buffer)	Poor general health Depression & severe mental illnesses	Semantic differential scale Medical records	LSOAs with a larger average garden size had lower rate of poor health (β=-1.938, SE 0.454), depression (β=-1.805, SE 0.424) and severe mental illnesses (β=-0.831, SE 0.966).	(+)
**Shape, pattern & connectivity (n=8)**						
Kim_2016 (n=92) [64]	Pattern of green space patches<br>* Fragmentation index (MPS)<br>* Shape irregularity index (MSI)<br>* Isolation index (MNN)	GIS analysis (805m buffer)	Quality of life	PedsQL	Children living in neighbourhoods with well-connected spatial patterns of urban forests and trees, as indicated by the indices MNN (β=0.608, p=0.001), were likely to had better quality of life. Fragmented or irregular green patches did not significant impact quality of life (β=-0.299 and -0.191 respectively, p>0.05).	(+)
Kim_2014 (n=61) [65]	Connectedness index (COHESION)	GIS analysis (805m buffer)	BMI	Clinically measured	Children living in neighbourhoods with well-connected landscape spatial patterns of urban forests and trees were likely to had lower BMI (β=-0.589, p=0.006).	(+)
Kim_2021 (n=2,301 census tracts) [67]	Configuration of tree canopy patches<br>* Size (TreeClus)<br>* Dispersion (TreeAgr)	GIS analysis	Asthma emergency visits	Medical records	Larger (β=-0.034, p<0.05 for TreeClus) and more aggregated (β=-0.034, p<0.05 for TreeAgr patches of trees, representing extended and connected canopy, were associated with fewer asthma emergency visits.	(+)
Sander_2017 (n=546 census blocks) [37]	Contiguity	GIS analysis	BMI	Self-measured height & weight	Recreational parks and conservation land contiguity was positively related to young-adult female BMI (β=0.562, p<0.05) and negatively related to older-adult female BMI (β=−0.508, p<0.05). Cemetery contiguity showed a positive relationship for middle-adult females (β=0.497, p<0.05)	(+)(–)
Wang_2019 (n=369 census tracts) [66]	Pattern of green space patches<br>* Fragmentation index (PD)<br>* Connectedness index (COHESION)<br>* Aggregation index (AI)<br>* Shape irregularity index (SHAPE_AM)	GIS analysis (805m buffer)	All-cause, cardiovascular, chronic respiratory and neoplasm mortality	Medical records	Census tracts with more connected, aggregated, coherent, and complex shape green spaces had a lower risk of all-cause mortality (COHESION: β=-0.259, 95% CI -0.329 to -0.190; AI: β=-0.085, 95% CI -0.119 to -0.052; SHAPE_AM: β=-0.039, 95% CI -0.056 to -0.023) and cause-specific mortality (β from -0.440 to -0.0372 across all indices, p<0.05).	(+)
Tsai_2016 (n=52 MSAs) [68]	Fragmentation of green space patches<br>* Aggregation (COHESION, ENN, PD)<br>* Contrast between patch types (ED, ECON)	GIS analysis	BMI	Self-reported height & weight	For forests and shrub lands, a greater edge contrast, which reflects increased possibility of access from other patch types (e.g. residential areas), was associated with lower BMI (β=0.370, p<0.01 and β=0.646, p<0.001, respectively). However, a higher density of shrub land (β=–0.504, p<0.01) and more connected patches of herbaceous vegetation (β=-0.281, p<0.05) were linked to higher BMI.	(+)(–)

Study*	Measure of quality	Tool(s) used to assess gren space quality**	Outcome	Tool(s) used to assess outcome **	Findings from study***	Direction of effect≠
Jaafari_2020 (n=87 hexagons) [69]	Pattern of green space patches<br>* Patch density (PD)<br>* Patch connectedness (COHESION)<br>* Shape irregularity (SHAPE-MN)	GIS analysis	Mortality of respiratory cancer diseases and respiratory diseases	Medical records	Mortality of respiratory diseases was reduced by green space via air pollution. Patch density (TE=0.302, p=0.007) and connectedness (TE=0.396, p<0.001) mediated this relationship.	(+)
Shen_2017 (n=48 districts) [70]	Pattern of green space patches<br>* Fragmentation index (PD)<br>* Aggregation indices (PLADJ, AI)<br>* Between-patch distance indices (ENN_MM, ENN_AM)	GIS analysis	Respiratory mortality	Medical records	Mortality of pneumonia and chronic lower respiratory diseases was reduced by minimizing fragmentation (total effect (TE)=0.112, p<0.05), and the mediation effects were mainly through reducing air pollutants rather than temperature. The mediation effects of between-patch distance (TE=0.027, p>0.05) and aggregation (TE=0.028, p>0.05) were non-significant.	(+)
**Safety (n=6)**						
Orstad_2020 (n=3,652) [71]	Perceived park crime	Dichotomous survey question	Mental health	Number of days with stress, depression, and emotion problems	Park proximity was indirectly associated with fewer days of poor mental health, mediated by park-based physical activity, but only among those not concerned about park crime (mediation index=0.04, 95% CI 0.01-0.10).	(+)
Camargo_2017 (n=1,392) [42]	Perceived safety of the way home	Semantic differential scale	Quality of life	EUROHIS-QOL 8-items	Perceived safety of the way home from the park significantly improves quality of life of park visitors (β=1.22, 95% 1.04-1.44).	(+)
Bai_2013 (n=893) [58]	Safety	Semantic differential scale	BMI	Self-reported	Perceived safety of parks was not significantly associated with BMI of residents (OR=1.03, 95% CI 0.83-1.29).	(o)
Pope_2018 (n=578) [59]	Safety	Dichotomous survey question	Psychological distress	GHQ-12	In a socially deprived neighbourhood, safety of green space was not significantly associated with reduced psychological distress (OR=0.84, 95% CI 0.56-1.27).	(o)
Tan_2019 (n=326) [45]	Perceived safety:<br>* Reduced visibility associated with dense vegetation<br>* Prospect of crime<br>* Presence of security guards<br>* Fear of falling<br>* Feeling unwell	Survey questionnaire (details unspecified)	Physical functioning, physical role, bodily pain and emotional role	SF-12v2	Perceived safety of urban green space has beneficial effects on role limitations due to physical problems (β=0.283, p<0.01) and bodily pain (β=0.204, p<0.05) among elderly residents in Hong Kong, but not emotional role limitations nor physical functioning (β=0.118 and 0.168 respectively, p>0.05). No beneficial effect was observed among Taiwan residents (β from 0.104 to 0.190, p>0.05).	(+)
Sugiyama_2009 (n=271) [60]	Safety:<br>*Night-time safety<br>*Safety along surrounding paths<br>*Lack of crime	Semantic differential scale	Health status<br>Quality of life	No. of days with poor physical/mental health<br>SWLS	Safety of neighbourhood open space significantly improved life satisfaction of elderly residents (OR=1.92, 95% CI 1.04-3.54), but did not have an impact on physical health (OR=1.33, 95% CI 0.69-2.55).	(+)
**Cleanliness and absence of incivilities (n=5)**						

Study*	Measure of quality	Tool(s) used to assess gren space quality**	Outcome	Tool(s) used to assess outcome **	Findings from study***	Direction of effect≠
Stark_2014 (n=44,282) [63]	Cleanliness score	Parks Inspection Program audit tool	BMI	Self-measured height & weight	Park cleanliness was associated with lower BMI of residents (β=-0.18, 95% CI -0.05 to -0.30).	(+)
Rundle_2013 (n=13,102) [56]	Weeds, litter, glass, graffiti score and overall cleanliness score	Parks Inspection Program audit tool	BMI	Clinically measured	Neither park cleanliness score (OR=-0.12, 95% CI -0.39 to 0.16) nor any of its components (OR=-0.34 to 0.22, p>0.05) was significantly associated with BMI.	(o)
Zhang_2019b (n=909) [43]	Aesthetics:<br>* Watered grass<br>* No graffiti<br>* No vandalism	POST	Quality of life	WHOQOL-BREF	Park aesthetics had no significant impact on all aspects of quality of life (physical psychological, social and environmental) in elderly residents (β from -0.031 to 0.087, p>0.05).	(o)
Bai_2013 (n=893) [58]	Cleanliness	Semantic differential scale	BMI	Self-measured height & weight	Perceived cleanliness of parks was not significantly associated with BMI of residents (OR=1.22, 95% CI 0.93-1.61).	(o)
Mears_2020b (n=345 LSOAs) [50]	Cleanliness	Environmental audits	Depression	Medical records	LSOAs with cleaner green space had lower rate of depression (β=-0.683, SE 0.295)	(+)
**Peacefulness (n=3)**						
Herranz-Pascual_2019 (n=137) [72]	Soundscape quality: unpleasant/pleasant, noisy/calm, stressful/relaxing, artificial/natural, monotonous/lively (vibrant), informative/uninformative and inappropriate/appropriate to surroundings	Semantic differential scale	Depression	Semantic differential scale	Different soundscape characteristics contributed differently to emotional restoration. Pleasant soundscape improved happiness (β=0.37, p<0.001), calmness (β=0.24, p<0.01), reduced anger (β=−0.21, p<0.01), sadness (β=-0.28, p<0.01) and stress (β=-0.35, p<0.001). Calming soundscape improved happiness (β=0.40, p<0.001), calmness (β=0.24, p<0.01) and reduced stress (β=-0.24, p<0.01). Fun soundscape improved happiness (β=0.30, p<0.001), calmness (β=0.23, p<0.01) and reduced stress (β=-0.23, p<0.01). Lively soundscape reduced sadness (β=-0.18, p<0.05). Natural soundscape improved happiness (β=0.32, p<0.001), calmness (β=0.25, p<0.01), reduced sadness (β=-0.20, p<0.05) and stress (β=-0.19, p<0.05).	(+)
Sugiyama_2009 (n=271) [60]	Nuisance<br>* Dogs and dog foulings<br>* Presence of young people	Semantic differential scale	Health status<br>Quality of life	No. of days with poor physical/mental health<br>SWLS	Reducing nuisance in neighbourhood open space did not have a significant impact on life satisfaction (1.07, 95% CI 0.58-2.00) nor physical health (OR=1.77, 95% CI 0.93-3.39) of elderly visitors.	(o)
Pazhouhanfar_2018 (n=250) [46]	Private environment	Semantic differential scale	Mood ratings (relaxed / happy / excited / calmed)	Semantic differential scale	Private environment was not a predictor of mood states of park visitors (β=0.02, p=0.78).	(o)
**Perceived quality / Satisfaction with quality (n=7)**						
Putra_2020 (n=4,969) [73]	Perceived quality by parents	Semantic differential scale	Prosocial behaviour	SDQ	Parental perceived green space quality was positively associated with prosocial behaviour in their children (β=0.20, 95% CI 0.13-0.27).	(+)

Study*	Measure of quality	Tool(s) used to assess gren space quality**	Outcome	Tool(s) used to assess outcome **	Findings from study***	Direction of effect≠
Feng_2018 (n=3,897) [74]	Perceived quality	Dichotomous survey question	Psychological distress; serious mental illnesses	K6-PDS	Symptoms of psychological distress (RR=0.89, 95% CI 0.85-0.93) and serious mental illnesses (OR=0.88, 95% CI 0.77-1.00) were fewer among postpartum women who considered local parks of good quality.	(+)
Feng_2019 (n=3,843) [75]	Perceived quality	Semantic differential scale	BMI	Self-measured height & weight	Compared to areas with the least green spaces (<5% coverage), areas with 21%–40% green space provided the most benefits in lowering postpartum BMI (β=-0.86, SE 0.33), regardless whether local parks were perceived good quality or not. Mothers in the greenest areas (>40%) only had statistically significantly lower BMI if they perceived local parks as high quality (β=−0.588, SE 0.431).	(+)
McEachan_2018 (n=805) [76]	Satisfaction with green space by parents	Semantic differential scale	Total difficulties, internalising difficulties, externalising difficulties & prosocial behaviours	SDQ	Among South Asian children, satisfaction with green space was significantly associated with fewer total behavioural difficulties (β=0.59, 95% CI -1.11 to -0.07), internalising behavioural difficulties (β=-0.28, 95% CI -0.56 to -0.003) and greater prosocial behaviour (β=0.20, 95% CI 0.02-0.38). No such associations were observed among Caucasian children.	(+)
Bai_2013 (n=893) [58]	Attractiveness	Semantic differential scale	BMI	Self-measured height & weight	Perceived attractiveness of parks was not associated with lower BMI among residents living near parks (OR=0.90, 95% CI 0.69-1.18).	(o)
Pazhouhanfar_2018 (n=250) [46]	Attractiveness	Semantic differential scale	Mood ratings (relaxed / happy / excited / calmed)	Semantic differential scale	Park attractiveness was not a predictor of mood states of park visitors (β=-0.03, p=0.70)	(o)
Jonker_2014 (n=N/A)	Satisfaction with quality	Semantic differential scale	Life expectancy & healthy life expectancy	National life table data	Satisfaction with quality of green space was associated with a modest increase in life expectancy (β=0.28, 95% CI 0.19-0.37) and healthy life expectancy (β=0.33, 95% CI 0.10-0.55).	(+)
**Combination of features (n=13)**						
Zhang_2017 (n=223) [77]	Perceived quality: recreational facilities, amenities, natural features, absent of civilities, accessibility, maintenance	Semantic differential scale	Neighbourhood satisfaction	Semantic differential scale	Perceived green space quality statistically mediated the link between the availability of accessible and usable green space and neighbourhood satisfaction (β=0.33, SE 0.09; mediation effect=0.16, 95% CI 95% CI=0.13-0.19). No direct effect was observed on wellbeing for either objective (β=0.49, SE 0.10) or perceived quality alone (β=0.40, SE 0.07).	(+)
Francis_2012 (n=911) [78]	Objective quality score: walking paths, shade, water features, irrigated lawn, birdlife, lighting, sporting facilities, playgrounds, type of surrounding roads, presence of nearby water<br>Subjective quality score: atmosphere, comfort, safety, attractiveness and maintenance, variety of things to do, presence of adequate seating, public art, other people	POST (objective)<br>Semantic differential scale (subjective)	Psychological distress	K6-PDS	High quality public open space was associated with reduced risk of psychosocial distress, but only with objective quality score (OR 2.46, 95% CI 1.42-4.27), not subjective score (OR=1.36, 95% CI 0.98-1.90).	(+)

Study*	Measure of quality	Tool(s) used to assess gren space quality**	Outcome	Tool(s) used to assess outcome **	Findings from study***	Direction of effect≠
Bird_2016 (n=380) [79]	Park typology: team sports features, pool-oriented features, perceived safety, cycling-oriented features, play area features, walking-oriented, aesthetically pleasing, incivilities, infrequent park installations, schoolyard features	Author-developed typology, with principal component analysis	% truncal fat	X-ray absorptiometry	Compared to control parks (low in team sports and play area features), children living near aesthetically pleasing parks with few team sports installations reported lower truncal fat percentage (β=−3.4, 95% CI -6.4 to -0.5). Other park typologies showed a non-significant trend towards reducing truncal fats (β from -1.8 to -0.2, p>0.1).	(+)
Kruize_2020 (n=3,947) [80]	Objective quality score: general characteristics, facilities, traffic safety, infrastructure, sidewalk amenities, incivilities<br>Satisfaction with green space: quality, amount, maintenance, safety	Environmental audits / Semantic differential scale	Mental wellbeing	MHI-5	Satisfaction with natural outdoor environment was a strong predictor of mental wellbeing (β=0.591, 95% CI 0.423-0.759) but objective streetscape quality was not (β=0.195, 95% CI −0.020-0.410).	(+)
Vries_2013 (n=1,641) [81]	Composite score: variation, maintenance, orderly arrangement, absence of litter, general impression	Semantic differential scale	Perceived general health; health complaints & mental health	SF-36; acute health-related complaint checklist; MHI-5	After accounting for effects of mediators, quality of streetscape greenery was associated with better perceived general health (β=0.090, SE 0.043), fewer acute health-related complaints (β=0.082, SE 0.043), and better mental health (β=1.164, SE 0.708).	(+)
Dillen_2012 (n=1,553) [82]	Green area quality: accessibility, maintenance, variation, naturalness, colourfulness, clear arrangement, shelter, absence of litter, safety, general impression	Environmental audits	Perceived general health; health complaints & mental health	SF-36; acute health-related complaint checklist; MHI-5	Quality of green areas was positively associated with higher perceived general health (β=0.189, SE 0.062) and fewer acute health-related complaints (β=-0.192, SE0.067), but not better mental health (β=2.278, SE1.267). Quality of streetscape greenery was positively associated with general health (β=0.165, SE0.044), health complaints (β=-0.156, SE0.049) and mental health (β=3.071, SE0.860).	(+)
Carter_2014 (n=440) [44]	Useability: in good conditions, well-equipped, including spaces to relax and socialise	Semantic differential scale	General health & vitality	SF-36v2	Residents who perceived green space to be highly useable were more likely to report good general health (OR=2.08, p=0.013) but not vitality (OR=1.68, p=0.068).	(+)
Dzhambov_2018 (n=399) [83]	Perceived quality: safety, maintenance, aesthetic, suitability for sport and social interactions, biodiversity	Semantic differential scale	Mental health	GHQ-12	Perceived green space quality provided indirect effects on mental health via pathways mediated by social cohesion and noise annoyance. However, the effects were only significant in single mediation model (social cohesion: β=-0.04, 95% CI -0.06 to -0.03; noise annoyance: β=-0.01, 95% CI -0.03 to -0.002) and parallel mediation model (social cohesion: β=-0.04, 95% CI -0.06 to -0.03); noise annoyance: β=-0.01, -0.03 to -0.002), not serial mediation model.	(+)
Tan_2019 (n=326) [45]	Aesthetics: colour, shape, diversity and seasonal variation of plants, maintenance, proportions of soft surfaces	Survey questionnaire (details unspecified)	Physical functioning	SF-12v2	Aesthetics of urban green space has no effect on physical and emotional health of elderly residents in both Taiwan and Hong Kong (β from -0.167 to 0.108, p>0.05).	(o)
Sugiyama_2009 (n=271) [60]	Pleasantness: adequacy for children to play, adequacy for adults to chat, variety of activities to engage in, quality of trees and plants, facilities (toilet, shelter)	Semantic differential scale	Health status<br>Quality of life	No. of days with poor physical/mental health<br>SWLS	Among older adults, pleasantness of neighbourhood open space significantly increased life satisfaction (OR= 1.94, 95% CI 1.03-3.63), but not physical health (OR=1.58, 95% CI 0.82-3.07).	(+)

Study*	Measure of quality	Tool(s) used to assess gren space quality**	Outcome	Tool(s) used to assess outcome **	Findings from study***	Direction of effect≠
Zhang_2019a (n=250) [84]	Visual sensation: Variety of plants, richness of plants' colour, plant light and shadow mottle, nice road texture, rich terrain, wide view, ornamental water<br>Auditory sensation: natural sound, sweet background music, happy people sounds (singing or playing instruments), quiet background, no traffic noise<br>Tactile sensation: road material is comfortable and the foot feels good, strong hydrophilic, seat is comfortable for sitting, comfortable grass for flat lay	Semantic differential scale	Restorative effect	Semantic differential scale	Visual sensory (β=0.214, 95% CI 0.046-0.405) and auditory sensation (β=0.524, 95% CI 0.304-0.773) were found to be linked with mental restoration directly, but tactile sensation was not (β=0.015, 95% CI −0.158-0.180). Mediation by behavioural activities and emotional response reduces the restorative effects of visual (β=0.170, 95% CI 0.053-0.320) and auditory sensations (β=0.150, 95% CI 0.063-0.299) but increased the effect by tactile sensation (β=0.184, 95% CI 0.079-0.307).	(+)
Mears_2020a (n=345 LSOAs) [61]	Quality<br>* Size ≥2 ha<br>* Predominantly natural feeling<br>* Good or better quality ratings from council assessment, based on: signage; provision of facilities; maintenance of paths; safety; planting and plant management; and cleanliness	Environmental audits	BMI	Clinically measured	LSOAs with access to more green spaces of good quality had lower obesity rates among year-6 students (β=-0.565, SE 0.269).	(+)
Mears_2020b (n=345 LSOAs) [50]	Quality<br>* Size ≥2 ha<br>* Predominantly natural feeling<br>* Good or better quality ratings from council assessment, based on: signage; provision of facilities; maintenance of paths; safety; planting and plant management; and cleanliness	Environmental audits	Poor general health	Semantic differential scale	LSOAs with access to better public green space did not have a lower rate of poor general health (β=-0.121, SE 0.177).	(o)

Notes:Within each quality domain, studies were arranged by study design, and then by sample size.

** Abbreviations: DA: Dissemination Areas; LSOA: Lower Layer Super Output Areas; MSA: Metropolitan Statistical Areas*

***Abbreviations: AMI: Acute myocardial infarction; ART: Attention Restoration Theory; BMI: body mass index; CES-D: Center for Epidemiologic Studies-Depression; CIDR: Comparative Illness and Disability Ratio; CVD: Cardiovascular diseases; EUROHIS-QOL-8: EUROHIS 8-item Quality of Life Questionnaire; GDS: Geriatric Depression Scale; GHQ-12: 12-item General Health Questionnaire; GIS: Geographic Information System; K10-PDS: Kessler 10-item Psychological Distress Scale; K6-PDS: Kessler 6-item Psychological Distress Scale; MHI-5: 5-item Mental Health Inventory; PANAS: Positive and Negative Affect Schedule; PedsQL: Paediatric Quality of Life Inventory; POST: Public Open Space Tool; POSDAT: Public Open Space Desktop Auditing Tool; PRS: Perceived Restorativeness Scale; PSS: Perceived Stress Scale; SDI: Shannon's Diversity Index; SDQ: Strengths and Difficulties Questionnaire; SF-8: 8-item Short Form Survey; SF-12: 12-Item Short Form Survey; SF-12v2: Short Form 12 item (version 2); SF-36: 36-Item Short Form Survey; SF-36v2: Short Form 36 item (version 2); SWLS: Satisfaction with Life Scale; WEMWBS: Warwick Edinburgh Mental Well-being Scale; WHOQOL-BREF: World Health Organization Quality-of-Life Scale*

**** Abbreviations: OR: Odds Ratio; pOR: Prevalence Odds Ratio; IHZ: Incidence Hazard Ratio; RR: Risk Ratio; pRR: Prevalence Risk Ratio; rs: Spearman's correlation coefficient; β: Regression coefficient; SE: standard error; SD: standard deviation*

≠ Notations for direction of change:

(+) Some evidence of protective associations;

(–) Some evidence of risk associations;

(o) No significant associations observed.
